# Caveolin1 and YAP drive mechanically induced mesothelial to mesenchymal transition and fibrosis

**DOI:** 10.1038/s41419-020-02822-1

**Published:** 2020-08-03

**Authors:** Raffaele Strippoli, Pilar Sandoval, Roberto Moreno-Vicente, Lucia Rossi, Cecilia Battistelli, Michela Terri, Lucía Pascual-Antón, Marta Loureiro, Francesca Matteini, Enrique Calvo, José Antonio Jiménez-Heffernan, Manuel José Gómez, Victor Jiménez-Jiménez, Fátima Sánchez-Cabo, Jesús Vázquez, Marco Tripodi, Manuel López-Cabrera, Miguel Ángel del Pozo

**Affiliations:** 1 Department of Molecular Medicine, Sapienza University of Rome, Viale Regina Elena 324, 00161 Rome, Italy; 2 National Institute for Infectious Diseases L. Spallanzani, IRCCS, Via Portuense, 292, 00149 Rome, Italy; 3 Mechanoadaptation & Caveolae Biology Lab, Cell and Developmental Biology Area, Centro Nacional de Investigaciones Cardiovasculares (CNIC), 28029 Madrid, Spain; 4 Programa de Homeostasis de Tejidos y Organos, Centro de Biología Molecular “Severo Ochoa”—CSIC, 28049 Madrid, Spain; 5 Cardiovascular Proteomics laboratory, Centro Nacional de Investigaciones Cardiovasculares Carlos III (CNIC) and CIBER Cardiovascular Diseases (CIBERCV), Instituto de Salud Carlos III, 28029 Madrid, Spain; 6 Departamento de Anatomía Patológica, Hospital Universitario La Princesa, Instituto de Investigación Sanitaria Princesa (IP), 28006 Madrid, Spain; 7 Bioinformatics Unit, Centro Nacional de Investigaciones Cardiovasculares Carlos III (CNIC), 28029 Madrid, Spain

**Keywords:** Mechanisms of disease, Chronic inflammation

## Abstract

Despite their emerging relevance to fully understand disease pathogenesis, we have as yet a poor understanding as to how biomechanical signals are integrated with specific biochemical pathways to determine cell behaviour. Mesothelial-to-mesenchymal transition (MMT) markers colocalized with TGF-β1-dependent signaling and yes-associated protein (YAP) activation across biopsies from different pathologies exhibiting peritoneal fibrosis, supporting mechanotransduction as a central driving component of these class of fibrotic lesions and its crosstalk with specific signaling pathways. Transcriptome and proteome profiling of the response of mesothelial cells (MCs) to linear cyclic stretch revealed molecular changes compatible with bona fide MMT, which (i) overlapped with established YAP target gene subsets, and were largely dependent on endogenous TGF-β1 signaling. Importantly, TGF-β1 blockade blunts the transcriptional upregulation of these gene signatures, but not the mechanical activation and nuclear translocation of YAP per se. We studied the role therein of caveolin-1 (CAV1), a plasma membrane mechanotransducer. Exposure of CAV1-deficient MCs to cyclic stretch led to a robust upregulation of MMT-related gene programs, which was blunted upon TGF-β1 inhibition. Conversely, CAV1 depletion enhanced both TGF-β1 and TGFBRI expression, whereas its re-expression blunted mechanical stretching-induced MMT. CAV1 genetic deficiency exacerbated MMT and adhesion formation in an experimental murine model of peritoneal ischaemic buttons. Taken together, these results support that CAV1-YAP/TAZ fine-tune the fibrotic response through the modulation of MMT, onto which TGF-β1-dependent signaling coordinately converges. Our findings reveal a cooperation between biomechanical and biochemical signals in the triggering of MMT, representing a novel potential opportunity to intervene mechanically induced disorders coursing with peritoneal fibrosis, such as post-surgical adhesions.

## Introduction

Biomechanical forces are increasingly recognized as major determinants of cell phenotype, and thus emerging drivers of numerous physiopathological processes. Biomechanical forces can be transmitted in cells through the interaction of different adhesion receptors such as integrins, cadherins, syndecans, CD44, and dystroglycan, with other cellular receptors or extracellular matrix (ECM) proteins^1–3^ as well as through changes in plasma membrane (PM) tension^4^, that can in turn be captured through specialized structures and membrane domains, including mechanosensitive ion channels and caveolae^1,5,6^. Caveolae are 60–80 nm diameter flask-shaped PM invaginations involved in numerous cellular processes including viral entry, vesicle transport, lipid metabolism, cell signaling, mechanosensing and mechanotransduction^7^. Caveolae, and particularly their essential component caveolin-1 (CAV1), contribute to sensing of and mechanoadaptation to mechanical stimuli, such as cell and substrate stiffness, cell stretching or loss of cellular substrate^6,8^ primarily by flattening and reorganizing their components upon changes of PM tension^9^. Caveolae are also linked to the actin cytoskeleton and align with stress fibers, thus potentially participating of a second layer of mechanosensing in the cell^10^. Caveolae-dependent mechanosensing orchestrates cell and tissue biomechanical adaptation, including the biochemical and mechanical remodelling of ECM^11,12^.

Mechanical cues promote adaptive cell responses, such as cytoskeletal remodeling and modulation of gene expression programs. Among pathways linking mechanical forces to gene expression of the transcriptional co-factor yes-associated protein (YAP) and its paralog WW domain-containing transcription regulator 1 (WWTR1/TAZ), key effectors of the Hippo signaling pathway emerge as master regulators integrating mechanotransduction with other cell functions^13^. YAP/TAZ activation and nuclear translocation upon increased ECM stiffness determines complex cellular responses^14,15^ through the regulation of broad gene expression programs mainly through interaction with TEA domain (TEAD) transcription factors to drive processes such as epithelial-to-mesenchymal transition (EMT) in tumors^16–18^.

Changes in biomechanical features of the extracellular matrix (ECM), such as ECM stiffness, can modify cell state and are major promoters of a fibrotic response^19^. Beyond ECM stiffness, sensing of mechanical stretching is characteristic of organs and tissues exposed to continuous variations of dynamic cues, such as respiratory and abdominal movements or the cyclic blood circulation pulse wave. In cells with epithelial features, the effects of cellular stretching have been analyzed especially on tissues composed of monocellular layers, such as lung epithelial cells and endothelium^20,21^. In this context, only a few studies to date have linked mechanical stretch to EMT^21–23^.

The peritoneum is lined by a monolayer of mesothelial cells (MCs) arranged on a basement membrane, which separates it from the submesothelial stroma. Mesothelial-to-mesenchymal transition (MMT) is a critical precursory event in the onset of peritoneal fibrosis during the practice of peritoneal dialysis (PD)^24,25^, in the formation of peritoneal adhesions (PAs) during abdominal surgery^4,26^, and in the accumulation of MC-derived carcinoma-associated fibroblasts during peritoneal metastasis^27^.

The onset of MMT and fibrosis depends on a complex reprogramming of MCs and other cell types involved. Fibrotic changes are mainly driven by cytokines of the transforming growth factor-β (TGF-β) superfamily. This process involves the induction of helix-loop-helix transcription factors, such as Snail, which directly inhibits the expression of epithelial markers including E-cadherin, and induces the expression of mesenchymal markers and ECM proteins such as collagens and fibronectin^28,29^.

We aimed to better understand the role of mechanical forces in fibrotic pathologies affecting peritoneum, and their underpinning molecular mechanisms. We found that exposure of MCs to cyclic mechanical stretch per se is sufficient to induce a bona fide MMT, being this induction largely dependent on endogenous TGF-β1 signaling. YAP/TAZ transcriptional activity sustains MMT-related changes. Additionally, CAV1, whose expression is regulated by YAP in MCs, limits MMT induction through the negative modulation of TGF-β1 signaling. Finally, a mouse peritoneal adhesion model demonstrated the relevance of CAV1 for curbing mechanical MMT and peritoneal fibrosis in vivo. We propose a model whereby mechanotransduction and biochemical signals (i.e. TGF-β1 signaling) engage in crosstalk to drive MMT induction and PA formation.

## Materials and methods

### Patient biopsies

A total of four parietal peritoneal biopsies corresponded to patients undergoing PD treatment, three adhesion biopsies were obtained after abdominal surgery and five ovarian cancer peritoneal implants were also evaluated. Control parietal peritoneal membrane samples were obtained from autopsy cases (*n* = 3). All tissue samples were fixed with neutral buffered formalin for 24 h and processed routinely for immunohistochemical analysis. The study was carried out in accordance with Good Clinical Practice guidelines and applicable regulations, as well as the ethical principles that have their origin in the Declaration of Helsinki. All included patients had signed informed consent forms and the study was approved by the Clinical Ethics Committee of Hospital Universitario Puerta de Hierro Majadahonda (ethic approval number: 11.17) (Madrid, Spain).

### Mice

CAV1-deficient mice and WT littermates (B6.Cg-CAV1tm1mls/J; ref. ^30^) were used for ex vivo and in vivo experiments. Mice from both genders were equally used in the different experimental conditions of ex vivo experiments, while exclusively male WT and CAV1^−/−^ mice were used for in vivo PA experiments. All animal procedures conformed to EU Directive 2010/63EU and Recommendation 2007/526/EC regarding the protection of animals used for experimental and other scientific purposes, enforced in Spanish law under Real Decreto 1201/2005, and were approved by the corresponding Animal Ethics Committee of Centro Nacional de Investigaciones Cardiovasculares Carlos III (CNIC) and Centro de Biología Molecular “Severo Ochoa” (CSIC).

### Cells

Mouse peritoneal MCs were obtained by digestion of parietal peritoneum samples from CAV1-deficient mice and WT littermates (B6.Cg-CAV1tm1mls/J). The samples were digested with a 0.125% trypsin solution containing 0.01% EDTA for 15 min with occasional agitation at 37 °C. Cells were cultured in DMEM F12 supplemented with 20% fetal calf serum, 50 U/ml penicillin, 50 μg/ml streptomycin, and 1% Biogro-2 (containing insulin, transferrin, ethanolamine, and putrescine) (Biological Industries, Beit Haemek, Israel). Biogro-2 is routinely added to favor in vitro MC growth, being washed out the day before performing the experiment. The purity of murine MC cultures was determined as in ref. ^12^.

Primary human peritoneal mesothelial cells (HPMCs) were obtained by digestion of omentum samples from patients undergoing abdominal surgery^31^. Written informed consent was obtained from omentum donors prior to elective surgery. The study was approved by the Clinical Ethics Committee of the Hospital Universitario de Princesa (Madrid, Spain). Omentum samples were digested with a 0.125% trypsin solution containing 0.01% EDTA for 15 min with occasional agitation at 37 °C. Cells were cultured in Earle’s M199 medium supplemented with 10% fetal calf serum, 50 U/ml penicillin, 50 μg/ml streptomycin, and 1% Biogro-2. The purity of HPMC cultures was determined by flow cytometry analysis of podoplanin expression (a standard mesothelial marker), and by ruling out any contamination with macrophages as determined by CD45 expression. HPMCs batches were >95% pure, and samples with >5% contaminating cells were discarded (Supplementary Fig. 1).

The human mesothelial cell line MeT5A (ATCC, Rockville, MD) was cultured in Earle’s M199 as above.

To induce MMT, HPMCs or MeT5A cells were stretched in a Flexcell^®^ FX-5000TM Tension System at 15% stretch, 0.86 Hz for 24 or 48 h. Plates covered with type I collagen (provided by the manufacturer) were used. Where indicated, cells were pretreated for 1 h with diluent or SB431542 (TGFBRI pharmacological inhibitor; final concentration: 5 μM) or with 1D11.16 (anti-TGFβ1 mAb; final concentration: 100 μg/ml).

### Antibodies and chemicals

Monoclonal antibodies against SNAI-1 (#3895), YAP (#14074) phospho-SMAD2/3 (#8828), SMAD2/3 (#8685), phospho-SMAD3 (#9520) and CAV1 (#3267) were from Cell Signaling Technology; monoclonal antibodies against α-SMA (#A2547) and pan-cytokeratin (#SRE0061) were from Sigma Aldrich (Saint Louis, MO); monoclonal anti GAPDH (#MAB374) was from Millipore (Burlington MA, USA); monoclonal anti-TEF-1 (sc-376113), polyclonal anti-RhoGDI (sc-360) and anti-CTGF (sc-14939) were from Santa Cruz Biotechnology; polyclonal anti-ZO-1 (#617-300) was from Zymed (Invitrogen, Carlsbad, CA); polyclonal anti-FSP1 (#A5114) was from Dako (Glostrup, Denmark); monoclonal anti-calretinin (#ab92341) was from Abcam; anti-MMP2 (#IM33) was from Merck (Darmstadt, Germany); monoclonal anti-human podoplanin (#566456) and FITC Mouse Anti-Human CD45 Clone HI30 (#55548) were from BD Bioscences (Franklin Lakes, NJ); secondary antibodies Alexa Fluor 488, 555, 574 and 594 (Thermofisher Scientific, Massachusetts, USA); Hoechst 33342 and 4,6-diamidino-2-phenylindole (DAPI) were from Invitrogen (Carlsbad, CA). SB431542 was from Selleck (Houston, TX). 1D11.16 anti-TGFβ1 mAb (BE0057) was from inVivoMab/Bio X Cell (Lebanon, NH).

### Western blotting

Cells were lysed in Laemmli buffer^32^, and Western blotting was performed as previously described^33^. Monolayers of MCs were lysed in modified RIPA buffer containing: 50 mM Tris-HCl, pH 7.4; 1% NP-40; 0.1% SDS; 0.25% Nadeoxycholate; 150 mM NaCl; 1 mM EDTA; 1 mM EGTA; 1 mM PMSF; 1 μg/ml each of aprotinin, leupeptin and pepstatin; and 25 mM NaF (all from Sigma). Equal amounts of protein were resolved by SDS-PAGE. Proteins were transferred to PVDF membranes (Millipore, Bedford, VA) and probed with antibodies using standard procedures. PVDF-bound antibodies were detected by chemiluminescence with ECL (Amersham Life Sciences, Little Chalfont, UK).

### FACS analysis

HPMC suspensions were preincubated with human Fc Receptors block (#564220 from BD Biosciences) to reduce nonspecific binding. Cells were stained with podoplanin-BV421 and CD45-FITC conjugated antibodies. Antibodies were omitted in negative controls. Flow cytometry data acquisition was performed on a FACSAria Fusion equipment (BD Biosciences). FlowJo software (Tree Star Inc., Ashland, OR) was used for data analysis.

### Enzyme-linked immunoassay

TGF-β1 concentration was determined by using standard enzyme-linked immunoassay (ELISA) kits (R&D Systems). Confluent HPMCs (200 × 10^3^) were left in static conditions or exposed to cyclic mechanical stretch for 24 h. Supernatants were collected and stored at −80 °C until their analysis. Results were represented as picograms per milliliter (pg/mL). Statistical significance was determined with a *t*-test with GraphPad Prism version 5.0 (La Jolla, CA, USA). Differences were considered significant at *p* < 0.05.).

### Confocal microscopy and immunofluorescence

Cells were fixed for 20 min in 3% formaldehyde in PBS, permeabilized in 0.2% Triton X‐100/PBS for 5 min, and blocked with 2% BSA for 20 min. Secondary antibodies (conjugated to Alexa-647, −488, or −541) were from Pierce Chemical Company (Rockford, IL, USA). Confocal images were acquired using a ZEISS LSM700 fitted with a ×20 and ×40 objective with a dipping lens. A PLAN-APOCHROMAT 25 × 0.8 Numerical Aperture objective was used. Suitable excitation lasers and emission filters were chosen to minimize bleedthrough between channels. Nucleus/cytoplasm distribution was estimated by measuring the intensity at a nuclear ROI, and at a ROI of equal size in the cytosol immediately adjacent to the nuclear region, as in Elosegui-Artola et al.^34^. Hoechst staining was used to delimit nuclear versus cytosolic ROIs. Statistical significance was determined with the nonparametric Mann–Whitney rank‐sum *U*‐test with GraphPad Prism version 5.0. Differences were considered statistically significant at *p* < 0.05.

### SiRNA-mediated knockdowns and lentiviral infection

siGENOME SMARTpool siRNAs against human YAP (L-012200-00-0005), TAZ (L-016083-00-0005); ON-TARGETplus and control siRNAs, were purchased from Dharmacon. To silence CAV1, a siRNA corresponding to human CAV1 (target sequence: AAGAGCTTCCTGATTGAGATT) plus two more sequences (CAV1 h46, GCUUCCUGAUUGAGAUUCAtt and CAV1 h48 CCUUCACUGUGACGAAAUAtt) or a control siRNA at the same concentration were used. siRNAs were transfected into cells using Lipofectamine^®^ RNAiMAX (Invitrogen) by reverse transfection as specified by the company. In all, 72 h after the last transfection, knockdown efficiency was determined by RT-qPCR and Western blot and cells were processed as indicated. MCs were also infected with lentiviruses encoding for pRR-CMV-Cav-IRES-GFP or empty vectors as controls as in ref. ^11^.

### Reverse-transcriptase polymerase chain reaction

RNA, extracted from cell cultures with ReliaPrep™ RNA Tissue Miniprep System (Promega, Madison, WI, USA), was reverse transcribed with iScriptTM c-DNA Synthesis Kit (Bio-Rad Laboratories, Inc., Hercules, CA, USA) according to the manufacturer’s instructions. cDNAs were amplified by qPCR reaction using GoTaq^®^ qPCR Master Mix (Promega, Madison, WI, USA). The specific primer pairs are listed in Suppl. Table 1. Relative amounts, obtained with 2^−ΔCt^ method, were normalized with respect to the housekeeping gene L32. Statistical significance was determined with a *t*-test with GraphPad Prism version 5.0 (La Jolla, CA, USA). Differences were considered significant at *p* < 0.05 (*p < 0.05; **p < 0.01; ***p < 0.001).

### RNAseq analysis

RNAseq data were analysed by the Bioinformatics Unit of the CNIC. Sequencing reads were pre-processed by means of a pipeline that used FastQC (http://www.bioinformatics.babraham.ac.uk/projects/fastqc/), to asses read quality, and Cutadapt v1.3^35^ to trim sequencing reads, eliminating Illumina adaptor remains, and to discard reads that were shorter than 30 bp. The resulting reads were mapped against reference transcriptome GRCm38.76 and quantified using RSEM v1.2.25^36^. Around 88% of the reads participated in at least one reported alignment. Expected expression counts calculated with RSEM were then processed with an analysis pipeline that used the Bioconductor package EdgeR^37^ for normalization (using TMM method) and differential expression testing. Changes in gene expression were considered significant if associated to Benjamini and Hochberg adjusted *p*-value < 0.05. Enrichment analyses were assayed using Ingenuity Pathway Analysis (IPA, Qiagen) and Gene Set Enrichment Analysis (GSEA, Broad Institute, Inc.) tools. Full RNAseq data are available through GEO accession number GSE146955 (https://www.ncbi.nlm.nih.gov/geo/query/acc.cgi?acc=GSE146955).

### Proteomics

#### Protein digestion and iTRAQ labeling

Protein concentration from matrix samples was measured by the Pierce BCA Protein Assay (Life Technologies, Thermo Scientific). For each sample, a total of 150ug of proteins were boiled in Laemmli buffer^32^ containing 50 mM DTT and run on a SDS-PAGE gel (10% resolving gel and 4% stacking gel) at 65 V. The electrophoresis was stopped when the front dye had barely passed into the resolving gel, ensuring concentration of all proteins into a unique band, and proteins were fixed during 30 min in a solution containing 10% (v/v) acetic acid and 40% (v/v) methanol. Staining was performed using GelCode^®^ Blue Stain Reagent (Thermo Scientific). Protein bands were excised from the gel, cut into cubes (2 × 2 mm) and treated with 55 mM iodoacetamide (IAM, SIGMA) to alkylate cysteine residues. In-gel digestion was performed overnight at 37 °C with modified porcine trypsin (Promega) at a final ratio of 1:10 (trypsin-protein) in 100 mM ammonium bicarbonate, pH 8.8. Digestion proceeded overnight at 37 °C. The resulting tryptic peptides were extracted twice by 1 h incubation at room temperature in 100% acetonitrile and 5% formic acid, dried-down and desalted onto C18 Oasis-HLB cartridges. Peptides were then subjected to isobaric labeling using the 4-plex iTRAQ Reagents Multiplex Kit (Sciex) as described before^38^. For increasing proteome coverage, iTRAQ-labeled samples were fractionated by cation exchange chromatography (Oasis HLB-MCX columns) into six fractions, which were desalted following the same procedure as explained above.

#### Protein identification and quantification

Tryptic peptide mixtures were subjected to nano-liquid chromatography coupled to mass spectrometry for protein identification and quantification. Peptides were separated on a C-18 reversed phase^39^ nano-column (75 µm I.D. and 50 cm, Acclaim PepMap100, Thermo Scientific) 13 and analyzed in a continuous acetonitrile gradient consisting of 0–30% B in 240 min, 50–90% B in 3 min (*B* = 90% acetonitrile, 0.5% acetic acid). A flow rate of ca. 200 nL/min was used to elute peptides from the RP nano-column to an emitter nanospray needle for real time ionization and peptide fragmentation on a Q-Exactive HF mass spectrometer (Thermo Fisher). An enhanced FTresolution spectrum (resolution = 70000) followed by the MS/MS spectra from most intense fifteen parent ions were analysed along the chromatographic run (272 min). Dynamic exclusion was set at 20 s.

Peptides were identified with Proteome Discoverer (version 2.1.0.81, Thermo Fisher Scientific) using SEQUEST-HT (Thermo Fisher Scientific). For database searching at the Uniprot database containing all sequences from human (May 18, 2016), parameters were selected as follows: trypsin digestion with 1 maximum missed cleavage sites and precursor and fragment mass tolerances of 800 ppm and 0.02 Da, respectively. Methionine oxidation was selected as dynamic modification, and Carbamidomethyl cysteine and N-terminal and Lys iTRAQ modifications were selected as dynamic modifications. Peptide identification was validated using the probability ratio method^40^ with an additional filtering for precursor mass tolerance of 10 ppm^41^. False discovery rate (FDR) was calculated using inverted databases and the refined method^42^. Protein quantification from reporter ion intensities and statistical analysis were performed using the SanXoT package^43^, based on the WSPP statistical model^44^. In this model, the accuracy of individual quantifications is taken into account by expressing protein abundance changes in units of standard deviation (log2-ratios), using the standardized variable Zq. The threshold for differential protein and peptide abundance was set at |Zq| ≥ 2.5. Alterations in biological functions as a consequence of the coordinated behavior of proteins, were analyzed by estimating functional category averages (Zc) according to the Systems Biology Triangle (SBT) model^45^. The quantified proteins were functionally annotated using the Ingenuity Knowledge Database (IPA) and DAVID. The DAVID repository includes 13 functional databases, including Gene Ontology, KEGG, and Panther. Categories with at least 5 protein components and |Zc| ≥ 1.5 were considered significantly differential and were subjected to cluster analysis. Proteomics raw data can be found in: ftp://PASS01527:TB853ht@ftp.peptideatlas.org/.

#### Peritoneal adhesion mouse model

A total of six CAV1^−/−^ and six WT male mice, 11–12 weeks old, were used in a standard surgical adhesion model as previously described^26^. Mice were anaesthetized with inhaled isoflurane during surgery. Briefly, an anterior midline incision was made through the abdominal wall and peritoneum. Three ischaemic buttons (IBs), spaced 1 cm apart, were created on the left side of the peritoneum by grasping 3 mm of the parietal tissue with a haemostat and ligating the base of each segment with 4–0 silk sutures. The incision was closed on both layers. The animals were sacrificed 5 days after surgery. Each animal received an adhesion score based on the number of IBs with attached adhesions. Extent (grade) and quality (tenacity) of adhesions were scored with a point scale (Supplementary Table 2)^26^. Both scores were averaged and never differed by more than 1. A meticulous search for other areas of adhesions inside the peritoneal cavity was also performed. Mice were scored in blind by two independent observers. IB tissue samples were fixed with 3.7% neutral-buffered formalin and processed routinely for histological, immunohistochemical and immunofluorencence studies. Statistical significance for all in vivo analysis was determined with the non-parametric Mann–Whitney rank-sum *U*-test with GraphPad Prism (La Jolla, CA, USA). Differences were considered significant at *p* < 0.05.

### Dual-immunofluorescence staining in mouse peritoneal tissues

Mouse peritoneal tissue sections (3 μm) were heated to expose the hidden antigens using Real Target Retrieval Solution containing citrate buffer, pH 6.0 (Dako). Samples are routinely pretreated with 50 mM NH4Cl solution to avoid formalin-related autofluorescence. Non-specific binding of secondary antibodies were blocked by pretreating slides with donkey serum (Abcam). Anti-pan-cytokeratin and anti-FSP1 antibodies were incubated at room temperature for 1 hour followed by Alexa Fluor 647 and 555 secondary antibodies. Finally, the slides were mounted with DAPI nuclear stain. Negative controls, in which primary antibodies were omitted, did not give rise to any detectable labeling. Images were captured with a LSM710 Zeiss Confocal Microscope (Zeiss, Germany).

### Immunohistochemistry

Immunohistochemical studies on patient peritoneal tissues were performed on serial sections 3 μm thick using a biotinylated secondary antibody followed by the R.T.U Vectastain Elite ABC Kit (Vector Laboratories, Burlingame, CA, USA). A similar method was applied for immunohistochemical studies in mouse tissues. The Vector M.O.M. Immunodetection Kit (Vector Laboratories, Burlingame, CA, USA) was applied according to the manufacturer’s instructions. All cases were revealed using DAB (Dako) as chromogen and finally counterstained with haematoxylin. Images were captured with a digital camera coupled to a brightfield microscope and 2–4 arbitrary fields (magnification ×200) for each sample were quantified using the analysis program Image-J 1.48 v (National Institute of Health, Bethesda, Maryland). Statistical significances were determined with the non-parametric Mann–Whitney rank-sum *U*-test with GraphPad Prism (La Jolla, CA, USA). Differences were considered significant at *p* < 0.05.

## Results

### Positive correlation among mechanotransduction, MMT progression and TGF-β1-related markers in peritoneal fibrosis patient biopsies

**Fig. 1:**
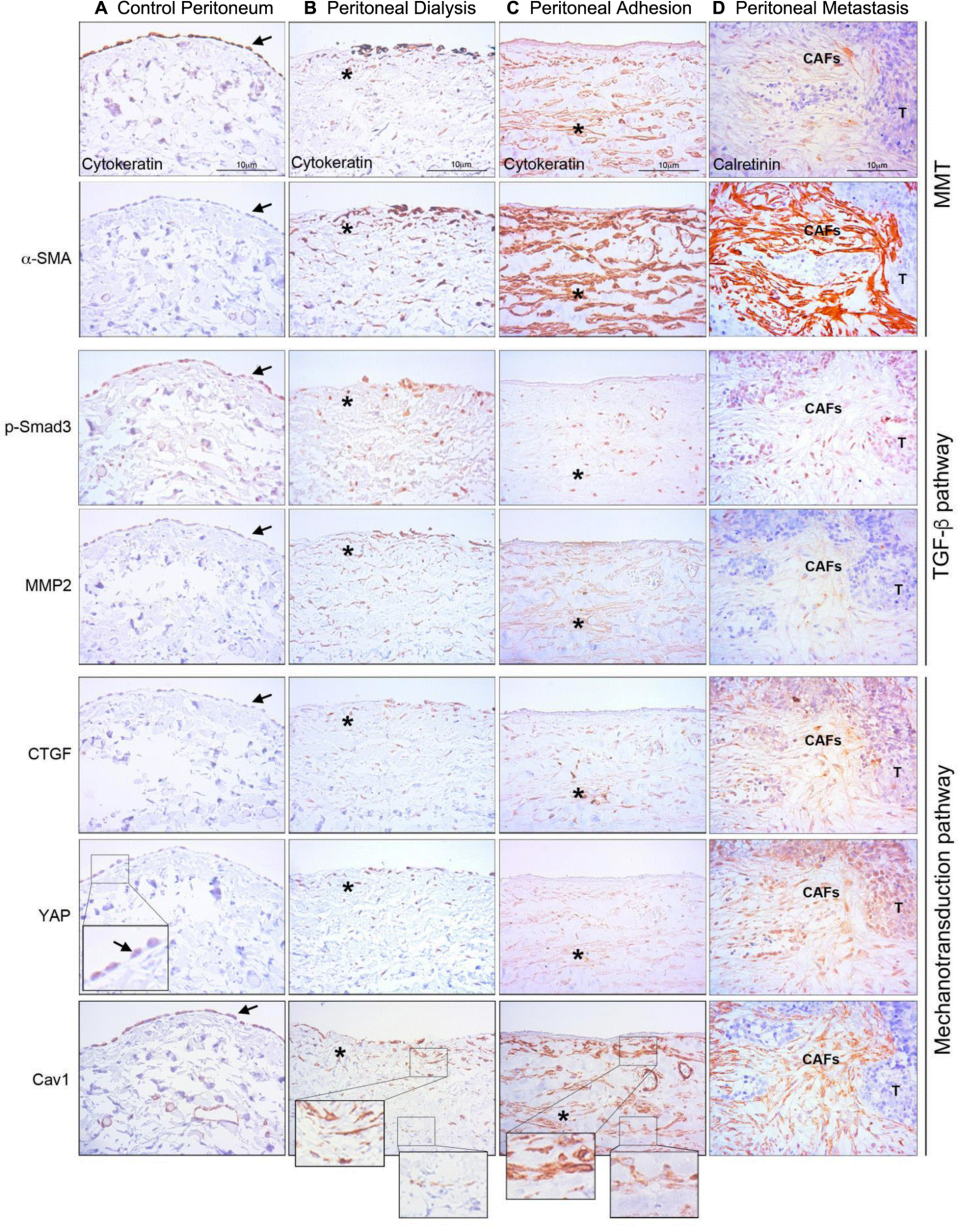
MMT-related biochemical and mechanical pathway markers in patient peritoneal biopsies. **a** Serial sections of a control peritoneal tissue show pan-cytokeratin staining confined to the preserved MC monolayer and absence of α-SMA expression. Mesothelial monolayer shows basal staining for phospo-Smad3 and MMP2, absence of CTGF staining, basal YAP (inset) and strong positivity for CAV1. **b** Serial sections of a fibrotic peritoneum from a patient undergoing PD. Pan-cytokeratin and α-SMA staining are detected at deeper levels of the peritoneum. MMT submesothelial area overlaps with nuclear phospho-Smad3, MMP2, CTGF, nuclear YAP and CAV1 staining. Of note, a decreased intensity of staining for CAV1 in submesothelial deeper zones can be appreciated as compared to upper areas (insets). **c** Serial sections of a post-surgical peritoneal adhesion show deeply located submesothelial cells stained for pan-cytokeratin, α-SMA, phospho-Smad3, MMP2, CTGF, YAP and CAV1. Of note, a decreased intensity of staining for CAV1 in submesothelial deeper zones can be appreciated as compared to upper areas (insets). **d** Serial sections of an ovarian cancer peritoneal implant show submesothelial accumulation areas of mesothelial-derived CAFs marked for calretinin and α-SMA. MMT-derived CAFs are positive for nuclear phospho-Smad3, MMP2, CTGF, nuclear YAP and CAV1. T tumor, CAFs carcinoma-associated fibroblasts, asterisk indicates overlapped staining areas.

Biopsies from (i) parietal peritoneum of patients undergoing PD^46^, PA development after abdominal surgery, or (iii) ovarian carcinoma peritoneal metastasis, were analyzed for the expression of markers related to mechanotransduction pathways (Hippo-YAP/TAZ and CAV1), as well as to TGF-β1 pathway as compared to healthy peritoneum samples. Serial sections of peritoneal fibrotic tissues showed immunohistochemical staining for mesothelial markers (pan-cytokeratin or calretinin) overlapping with α-SMA (a myofibroblast marker) in submesothelial areas indicating an accumulation of mesothelial-derived myofibroblasts via MMT. Accordingly, MMT-derived myofibroblasts expressed phospho-SMAD3 and MMP2, indicative of TGF-β1 signaling-activation. Additionally, the staining for CTGF, a YAP-driven molecule, and for YAP overlapped with MMT-positive areas, suggesting a role for YAP/TAZ-driven programmes in MMT. Of note, basal partial nuclear translocation of YAP could be observed in the mesothelial monolayer of control biopsies. CAV1, a membrane mechanotransducer, was expressed in the MC monolayer lining the surface of healthy samples, as well as in the submesothelial myofibroblasts of the three considered fibrotic pathologies. Interestingly, a relative decrease in CAV1 staining could be observed in submesothelial deeper zones of PD and PA patients, as compared to upper areas. The presence of both biochemical and mechanical MMT-associated markers was constant in the series of patient biopsies included in this study (Fig. 1). These results suggest the existence of an inter-relation between biochemical (TGF-β1-induced) and biomechanical (YAP/TAZ and CAV1-mediated) signals during the pathogenesis of peritoneal fibrotic pathologies.

### RNA-sequencing and quantitative proteomics analyses of mesothelial cells reveal bona fide MMT signatures as integral to adaptation to cyclic stretching

**Fig. 2:**
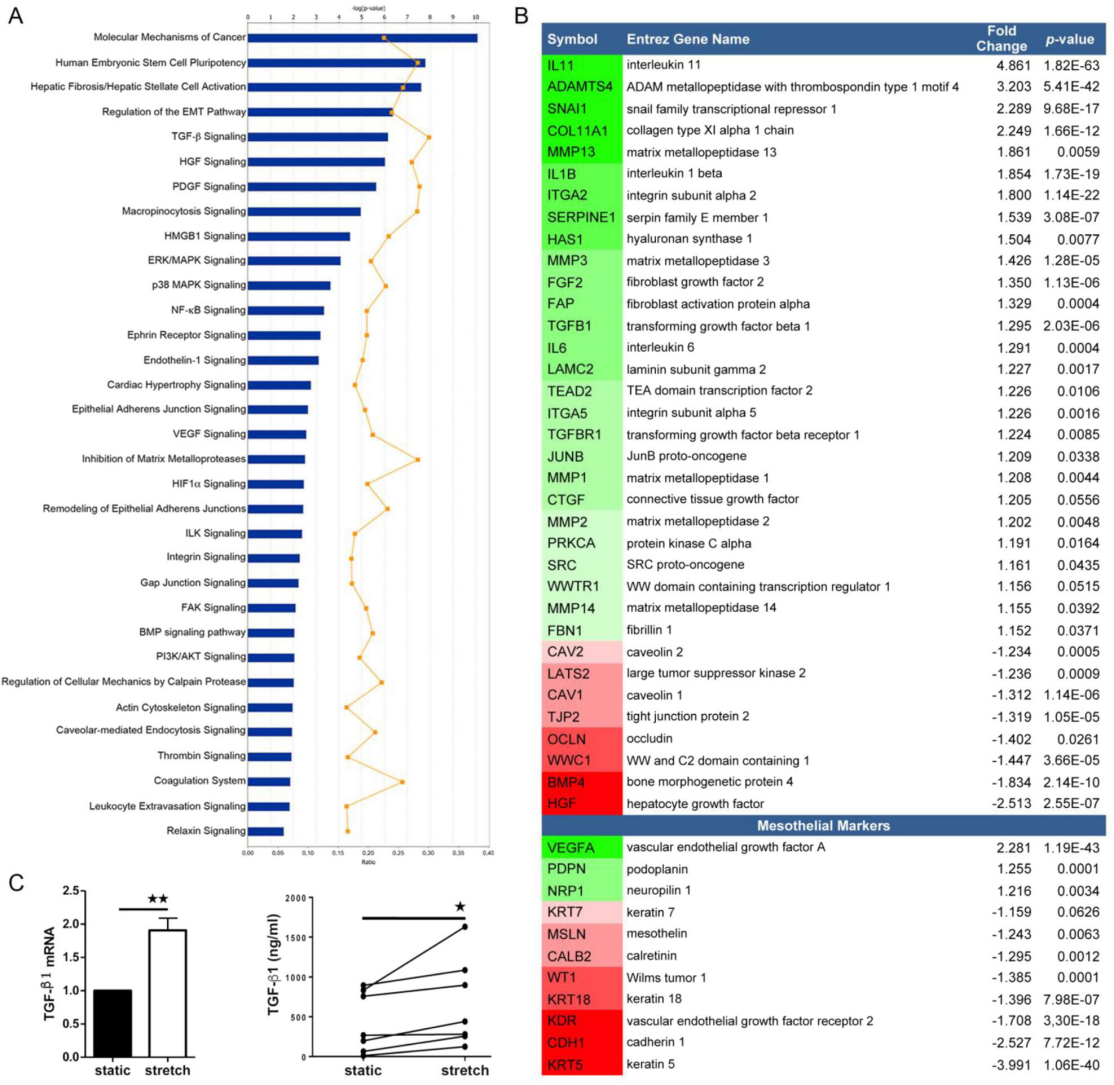
RNA-sequencing analysis of HPMCs exposed to cyclic mechanical stretch during 24 h. **a** Differentially regulated MMT-related canonical pathways analyzed with IPA software. The upper horizontal axis indicates statistical significance, corrected with the method of Benjamini–Hochberg. **b** Subset of genes significantly dysregulated upon stretching conditions (*n* = 3) as compared to static (*n* = 3). Specific mesothelial cell genes are listed at the bottom. Genes upregulated by exposure to mechanical stretch are in green, whereas those downregulated are in red. **c** qRT-PCR from total lysates (left) and ELISA assay from supernatants (right) of TGF-β1 of HPMCs exposed to cyclic mechanical stretch for 24 h (*n* = 7).

In order to obtain a whole picture of the changes taking place during exposure of mesothelial cells to intraperitoneal biomechanical forces, we profiled transcriptomic changes induced by cyclic mechanical stretch on primary human MCs. 1308 genes were upregulated and 1390 genes were downregulated in MCs upon cyclic stretching as compared to unstimulated MCs (*p*-value ≤ 0.05). Upon querying these gene subsets for Ingenuity Pathway Analysis (IPA), we found several entries related to cancer mechanisms and fibrotic disorders as well as MMT-related pathways, (Fig. 2a). Detailed interrogation across MMT-related gene subsets revealed an upregulation of mesenchymal genes, as well as the repression of epithelial-related markers. In particular, Snail family transcriptional repressor 1 (SNAI1), as well as profibrotic, proinflammatory and proangiogenic factors, including TGF-β1, IL-11, IL-6 were robustly induced by cyclic stretching. Interestingly, the expression levels of caveolins CAV1 and CAV2 were significantly reduced. A list of mesothelium-specific markers was created on the basis of published transcriptomic studies from our group^27,39^. Variation of expression of these genes showed induction of MMT upon exposure to mechanical stretch similarly to MMT induced upon exposure to PD fluid or during peritoneal metastasis^47,48^ (Fig. 2b and Supplementary Table 3).

An additional independent analysis using Gene Set Enrichment Analysis (GSEA) software recapitulated these results (Supplementary Fig. 2A–C). Moreover, GSEA analysis showed a significant upregulation of CAV1 negatively regulated targets, and the complementary downregulation of CAV1 positively regulated genes, in stretching with respect to static conditions (Supplementary Fig. 2B, C). Finally, network reconstruction with IPA revealed that many of these genes were dependent on, or connected to, TGF-β1 expression, consistent with a significantly upregulated TGF-β1 signaling gene signature (Fig. 2b).

**Fig. 3:**
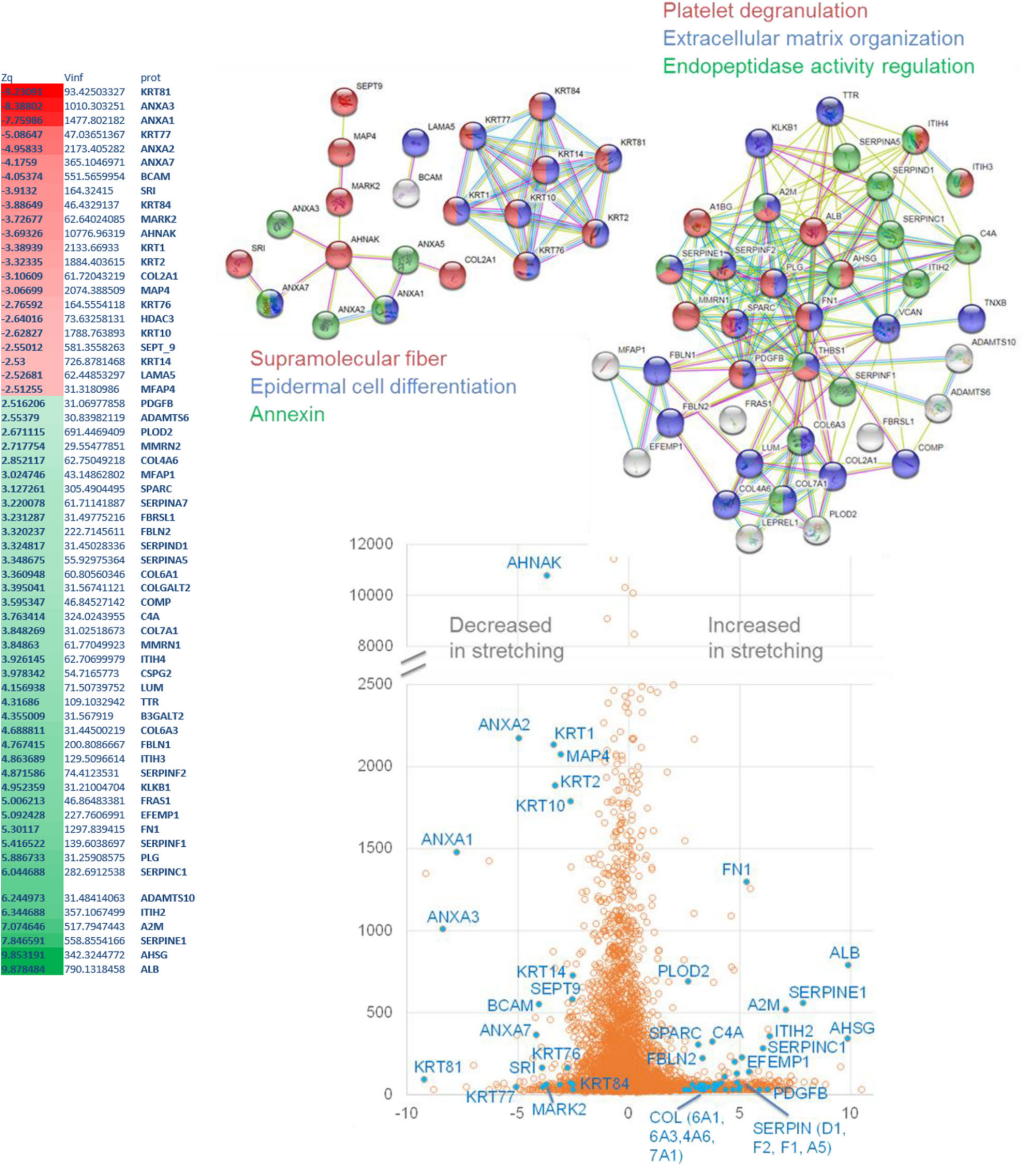
High throughput quantitative proteomics analysis of MeT5A cells exposed to cyclic mechanical stretch for 48 h. Significantly differentially regulated MMT-related proteins are listed in the top left table. Negative Zq values (red) represent a decrease in cells exposed to mechanical stretch as compared to static conditions. Positive Zq values (green) represent an increase in cells exposed to mechanical stretch as compared to static conditions. Top Right, STRING analysis of proteins significantly induced or downregulated and description of functional interactions. Bottom, the points represent distribution of corrected log2 ratios (static/stretch) (Xq-X) of protein quantifications according to their statistical weight (Wq, a parameter that measures the accuracy of protein quantifications, according to the WSPP model (Navarro et al.^44^)).

When surveying our datasets for YAP/TAZ-associated signatures, we observed broad transcriptional changes for members of YAP/TAZ regulatory networks, in cells subject to cyclic stretching as compared to unstimulated cells. Importantly, genes specifically regulated by both YAP/TAZ and TGF-β1, such as CTGF and SERPINE1, were significantly upregulated upon cyclic stretching (Fig. 2b) suggesting a cooperation between both pathways. Our transcriptomic data and network analysis also suggested a relevant role for caveolae in mechanically induced MMT (Supplementary Fig. 3). TGF-β1 increased expression in MCs exposed to mechanical stretching was confirmed by RT-qPCR and by ELISA (Fig. 2c). Quantitative proteomics analysis further confirmed our interpretation that MMT signatures are upregulated upon mechanical stretching, revealing a significant increase in abundance of collagens, fibronectin and serpins, as well as of numerous proteins related to the TGF-β pathway; and a robust reduction in epithelial markers such as cytokeratins (Fig. 3).

Taken together, these data support that exposure to cyclic mechanical stretch is sufficient to promote a bona fide MMT and suggest an interplay between biomechanical (YAP/TAZ and CAV1 pathways) and biochemical (TGF-β1 pathway) signals.

### Cooperation between YAP/TAZ-biomechanical and TGF-β1-biochemical pathways during MMT program

**Fig. 4:**
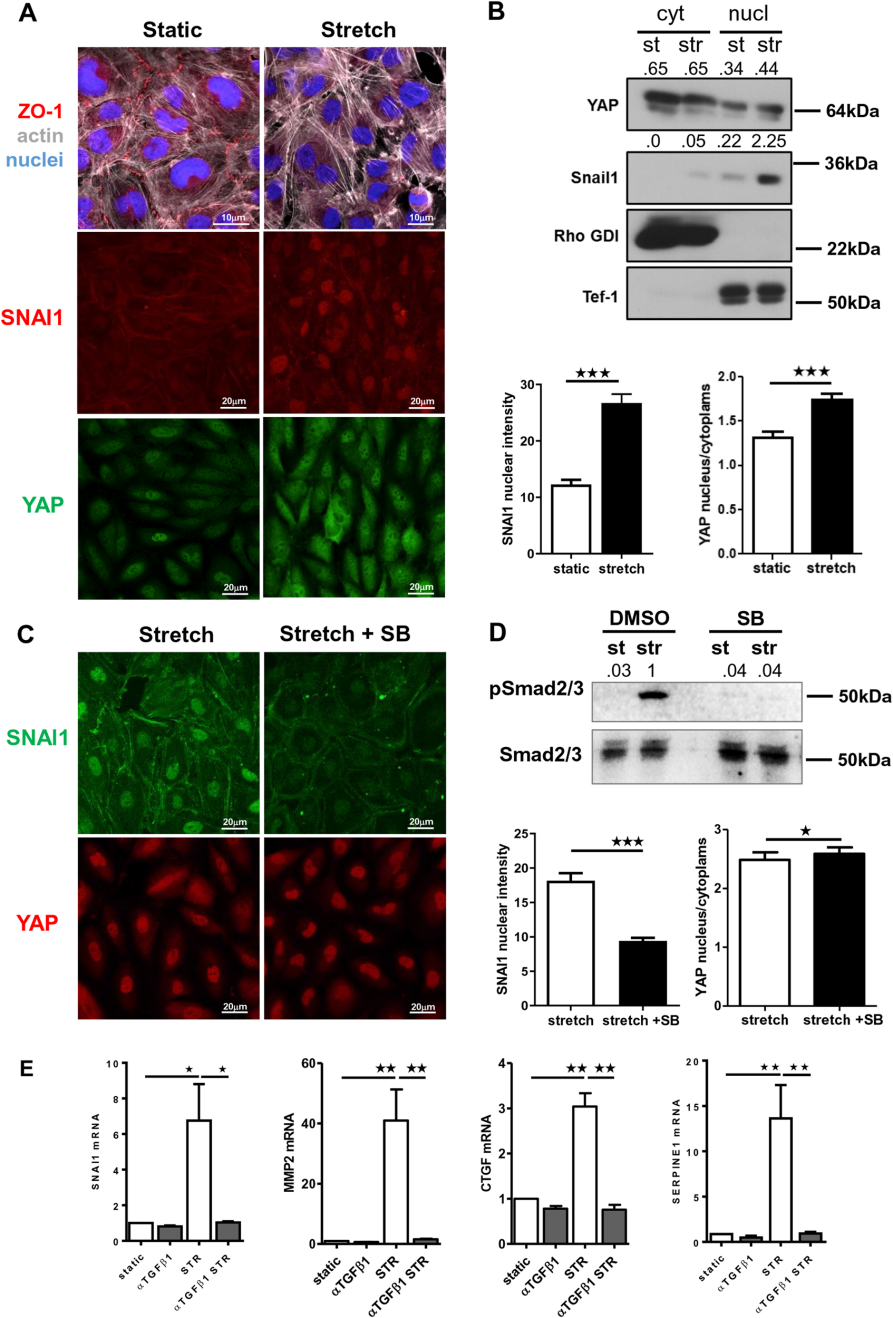
Exposure to cyclic mechanical stretch induces a bona fide MMT. **a** Immunofluorescence of HPMCs exposed for 24 h to a cyclic mechanical stretch or maintained in static conditions. Fixed cells were stained with monoclonal antibodies against ZO-1, SNAI1, YAP, and Alexa-647-conjugated phalloidin. Nuclei were stained with Hoechst. Images are representative of three independent experiments. Right, quantification of nuclear intensity. Histograms show the mean fluorescence intensity of nuclear SNAIl or nuclear/cytoplasmic YAP staining quantified using ImageJ analysis software. Bars represent the mean ± SEM. At least, a total of 150 cells were analyzed per condition. **b** Western blot analysis of YAP and SNAI1 subcellular distribution in HPMCs maintained in static conditions or exposed to cyclic mechanical stretch for 48 h. RHO-GDI and Tef-1 were used as cytosolic and nuclear markers, respectively. Representative experiment of three performed. **c** Left. effect of TGF-β1 inhibition on SNAI1 and YAP expression. MCs were pretreated with SB431542 (5 μM) for 1 h and then left untreated or exposed to mechanical stretch for 24 h. Fixed cells were immunostained for SNAI1 and YAP. Nuclei were stained with Hoechst 33342. **c** Right; quantification of nuclear intensity (for SNAI1) or nuclear/cytoplasmic intensity (for YAP). Histograms show mean fluorescence intensity quantified using the ImageJ software. Bars represent the mean ± SEM. At least, a total of 150 cells were analyzed per condition. **d** Western blot expression of phospho-SMAD2/3 and total SMAD2/3 in HPMCs exposed to cyclic mechanical stretch. Cells were left untreated or pretreated with SB431542 (5μM) for 1 h and then left untreated or exposed to mechanical stretch for 24 h. **e** Quantitative RT-PCR expression analysis of SNAI1, MMP2, CTGF, SERPINE1 in MeT5A cells exposed to cyclic mechanical stretch for 48 h. Cells were left untreated or pretreated with anti-TGF-β1 monoclonal antibody 1D11.16 (100 μg/ml). Quantitative RT-PCR was performed on total RNA. L34 mRNA levels were used for normalization. Bars represent the mean ± SEM of duplicate determinations in four independent experiments.

The induction of MMT upon exposure to mechanical stretch was confirmed by confocal microscopy experiments. Exposure of MCs to cyclic mechanical stretching for 48 h induced morphological and molecular changes, such as acquisition of a fibroblast-like phenotype and loss of zonula occludens 1 (ZO-1) at cellular junctions. At the same time, a significant increase in SNAI1 nuclear staining and YAP nuclear translocation was observed in stretching conditions (Fig. 4a). Nucleus/cytoplasm separation confirmed the upregulation and nuclear localization of SNAI1, as well as increased nuclear translocation of YAP as a result of mechanical stimulation (Fig. 4b). Of note, we detected partial YAP nuclear translocation in unstimulated cells (Fig. 4a), in accordance with our previous observations on healthy peritoneum (Fig. 1). These data confirm that mechanically induced MMT is associated with an activation of YAP/TAZ and TGFβ1/SNAI1 pathways.

In order to verify a causal role of TGF-β1 in MMT–like changes induced by mechanical stretch and its implication in the activation of YAP pathway, TGF-β1 activity was inhibited either using SB431542 (a TGF-β receptor (TGFBRI) pharmacological inhibitor) or 1D11.16 (a TGF-β1-blocking monoclonal antibody). Importantly, treatment with SB431542 abolished the induction of SNAI1 upon exposure to mechanical stretching, as well as SMAD2/3 phosphorylation, while inducing a slight increase in YAP nuclear/cytoplasmic localization (Fig. 4c, d). When analyzing the role of TGF-β1 inhibition on target gene expression, we found that both genes dependent on TGF-β pathway (SNAI1 and MMP2) and YAP/TAZ gene targets (CTGF and SERPINE1) were significantly blocked by the 1D11.16 antibody (Fig. 4e).

**Fig. 5:**
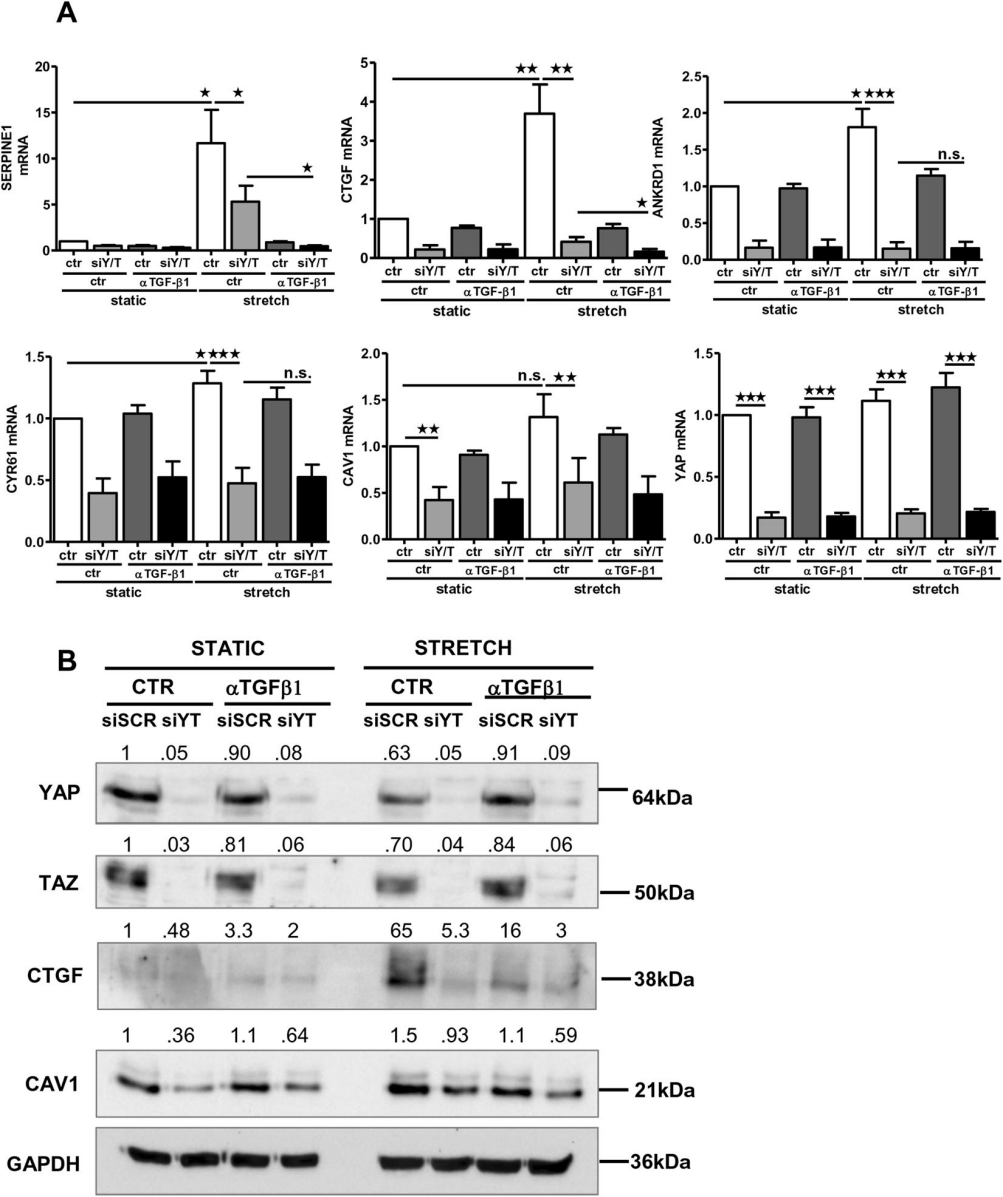
Cooperation between YAP/TAZ and TGF-β1 signaling in the expression of relevant genes for MMT program. **a** qRT-PCR analysis of SERPINE1, CTGF, ANKRD1, CYR61, CAV1 and YAP expression in MeT5A cells exposed to cyclic mechanical stretch for 48 h. Cells were transfected with control or YAP/TAZ-targeting siRNAs. After transfection, cells were left untreated or pretreated with anti-TGFβ1 monoclonal antibody (100 μg/ml) and then exposed to cyclic mechanical stretch for 48 h. Quantitative RT-PCR was performed on total RNA. L34 mRNA levels were used for normalization. Bars represent the mean ± SEM of duplicate determinations in four independent experiments. **b** Cells were treated as in **a**. Western blot analysis was performed on total lysates. GAPDH was detected as a loading control. Data are representative of three independent experiments.

The functional role of YAP/TAZ was then evaluated by siRNA-mediated knockdown. In accordance with results shown above, YAP/TAZ silencing led to downregulation of its target genes SERPINE1, CTGF, ANKRD1 and CYR61, and a blunting of their induction by exposure to mechanical stretch. Interestingly, TGFβ1 inhibition synergized with YAP/TAZ silencing in inhibiting a subset of genes (SERPINE1 and CTGF) but not ANKRD1 and CYR61, genes whose expression appears to be more dependent of YAP/TAZ activity. Of note, CAV1 expression was dependent of YAP/TAZ (Fig. 5a). Expression changes of CTGF, CAV1 and YAP were confirmed by Western Blot analysis (Fig. 5b).

These results demonstrate a direct implication of a TGF-β1-dependent biochemical pathway in mechanically induced MMT, and suggest a cooperation with YAP/TAZ-dependent mechanotransduction for the induction of a bona fide MMT.

### Lack of CAV1 exacerbates changes induced by mechanical stretching through activation of TGF-β1 pathway

**Fig. 6:**
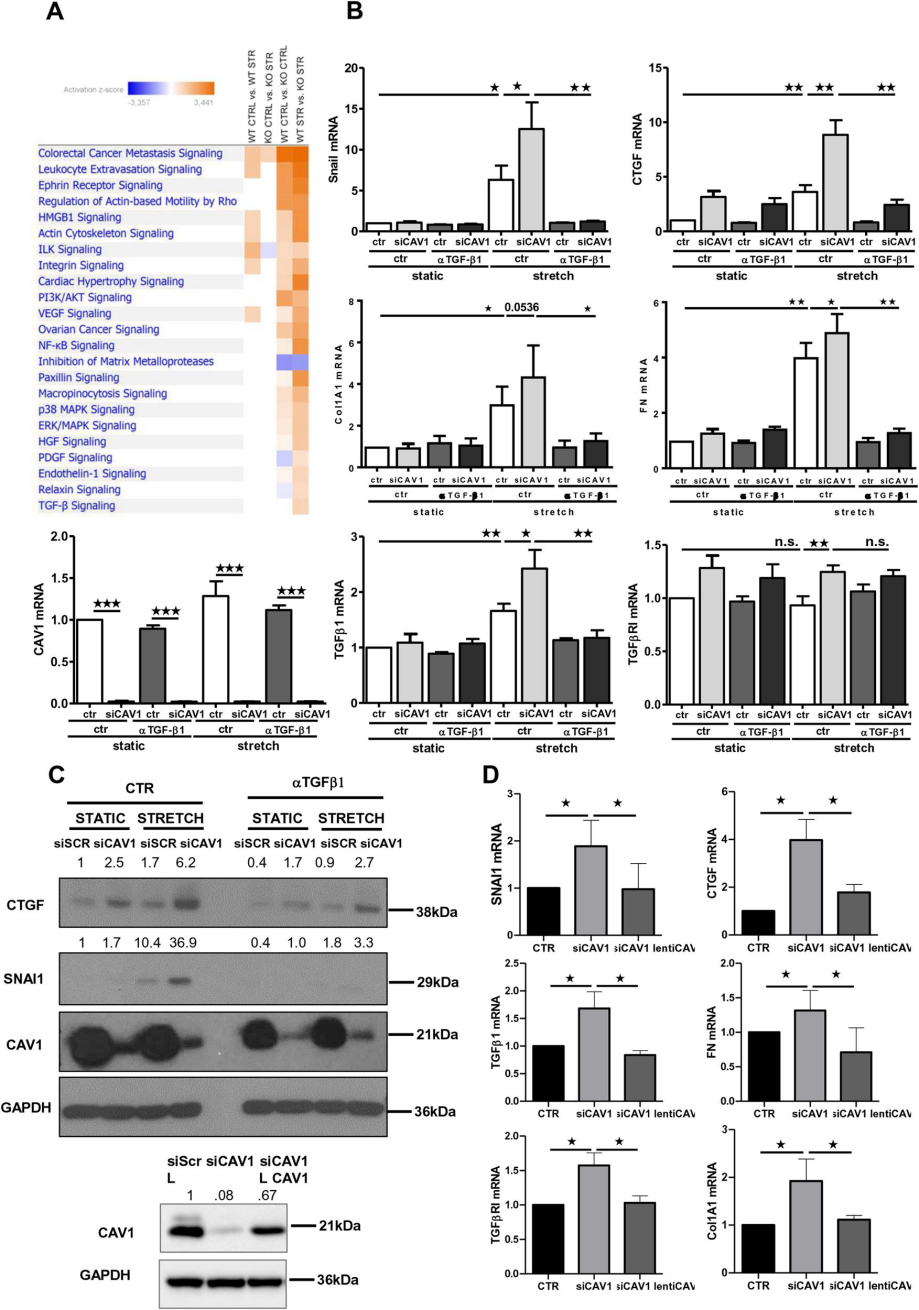
MMT-like phenotype in CAV1-deficient mouse and human MCs upon exposure to mechanical stretch is dependent on endogenous TGF-β1 production. **a** Comparative view of IPA enrichment results after RNA-sequencing analysis of primary mouse MCs isolated from WT and CAV1^−/−^ mice, upon static (*n* = 3) or stretching conditions (*n* = 3) for 24 h. The heatmap presents z-score values for WT control vs. WT stretch, KO control vs. KO stretch, WT control vs. KO control and WT stretch vs. KO stretch. Orange and blue colors, representing positive and negative z-scores, respectively, indicate the predicted state in the first vs. the second condition. *p*-values were corrected with the Benjamini–Hochberg method. **b** qRT-PCR analysis of SNAI1, CTGF, FN, Col1A1, TGFβ1, TGFBR1 and CAV1 expression in MeT5A cells exposed to cyclic mechanical stretch for 48 h. Cells were transfected with control or CAV1-targeting siRNAs. After transfection, cells were left untreated or pretreated with α-TGFβ1 (100 μg/ml) and then cells exposed to cyclic mechanical stretch for 48 h. Quantitative RT-PCR was performed on total RNA. L34 mRNA levels were used for normalization. Bars represent the mean ± SEM of duplicate determinations in four independent experiments. **C** MCs were treated as in **a**. Western blot analysis was performed on total lysates. GAPDH was detected as a loading control. Data are representative of three independent experiments. **d** qRT-PCR analysis of SNAI1, CTGF, FN, Col1A1, TGFβ1, and TGFBR1 expression in MeT5A cells. Cells were first transfected with control (siScr) or CAV1-targeting siRNA (siCAV1) and exposed to mechanical stretch for 48 h. Cells were then infected with control (L) or lentivirally encoded CAV1 (L CAV1) and maintained under mechanical stretch for other 48 h. Bars represent mean ± SEM in five independent experiments. CAV1 expression by WB is shown in the left. GAPDH was detected as a loading control.

CAV1 has recently emerged as a pivotal regulator of YAP/TAZ-dependent mechanotransduction^5,6^. In order to infer a potential role for CAV1 during mechanically induced MMT, RNAseq was performed on primary MCs isolated from WT and CAV1^−/−^ mice upon static or stretching conditions. We identified a total of 174 differentially expressed genes in WT static vs. WT stretch dataset (*p*-value ≤ 0.05); 183 in CAV1^−/−^ static vs. CAV1^−/−^ stretch dataset (*p*-value ≤ 0.05); 1153 dysregulated genes in WT vs. CAV1^−/−^ dataset in static conditions (*p*-value ≤ 0.05) and 989 in WT vs. CAV1^−/−^ dataset upon stretching (*p*-value ≤ 0.05) (Fig. 6a). Stretching-induced bona fide MMT gene signature observed in human RNAseq (Fig. 2) was recapitulated on wild-type mouse MCs, since we observed an enrichment in genes mapping to functional annotation categories related to MMT among gene subsets significantly activated by mechanical stretching. Interestingly, non-stretched CAV1^−/−^ MCs comparatively exhibited a mesenchyme-like phenotype, characterized by significant changes in the expression of gene programs enriched in several of those same functional categories. In addition, a significant activation of TGF-β signaling was detected in CAV1^−/−^ MCs under stretching conditions as compared to WT, suggesting that CAV1 exerts a negative regulation of TGF-β1 activation during mechanical stretch (Fig. 6a). Accordingly, a detailed analysis of specific genes showed an exacerbation of stretching-induced MMT gene expression signatures in CAV1^−/−^ MCs as compared to wild type MCs. Network analysis identified CAV1 and TGF-β1 as central nodes onto which modules enriched in integrins and MMPs, and proinflammatory and proangiogenic mediators, respectively, converge (Suppl. Fig. 4 and Supplemnetary Table 4).

To explore the roles of CAV1 and TGF-β1 during mechanical stretch-induced MMT in a human experimental model, CAV1 was knocked-down with a specific siRNA in human MCs. CAV1 depletion led to a significant increase of the expression of mesenchymal genes, such as SNAI1 and CTGF, as well as ECM genes, FN and COL1A1, upon exposure to stretching (Fig. 6b). The use of two additional specific sequences led to similar statistically significant changes (Supplementary Fig. 5). Expression changes of SNAI1 and CTGF were further confirmed by WB analysis (Fig. 6c). Additionally, CAV1 depletion upregulated the expression of both TGF-β1 and TGFΒR1. Importantly, the induction of mesenchymal genes associated with CAV1 depletion during exposure to mechanical stretch was dependent on TGF-β1 signaling, since it was significantly reduced by the anti-TGF-β1 monoclonal antibody 1D11.16 (Fig. 6d).

Taken together, these results indicate that CAV1 deficiency positively regulates MMT induction upon exposure to mechanical stretching, at least in part through hyperactivation of the TGF-β1 pathway.

### CAV1^−/−^ mice show increased MMT and peritoneal adhesion formation

**Fig. 7:**
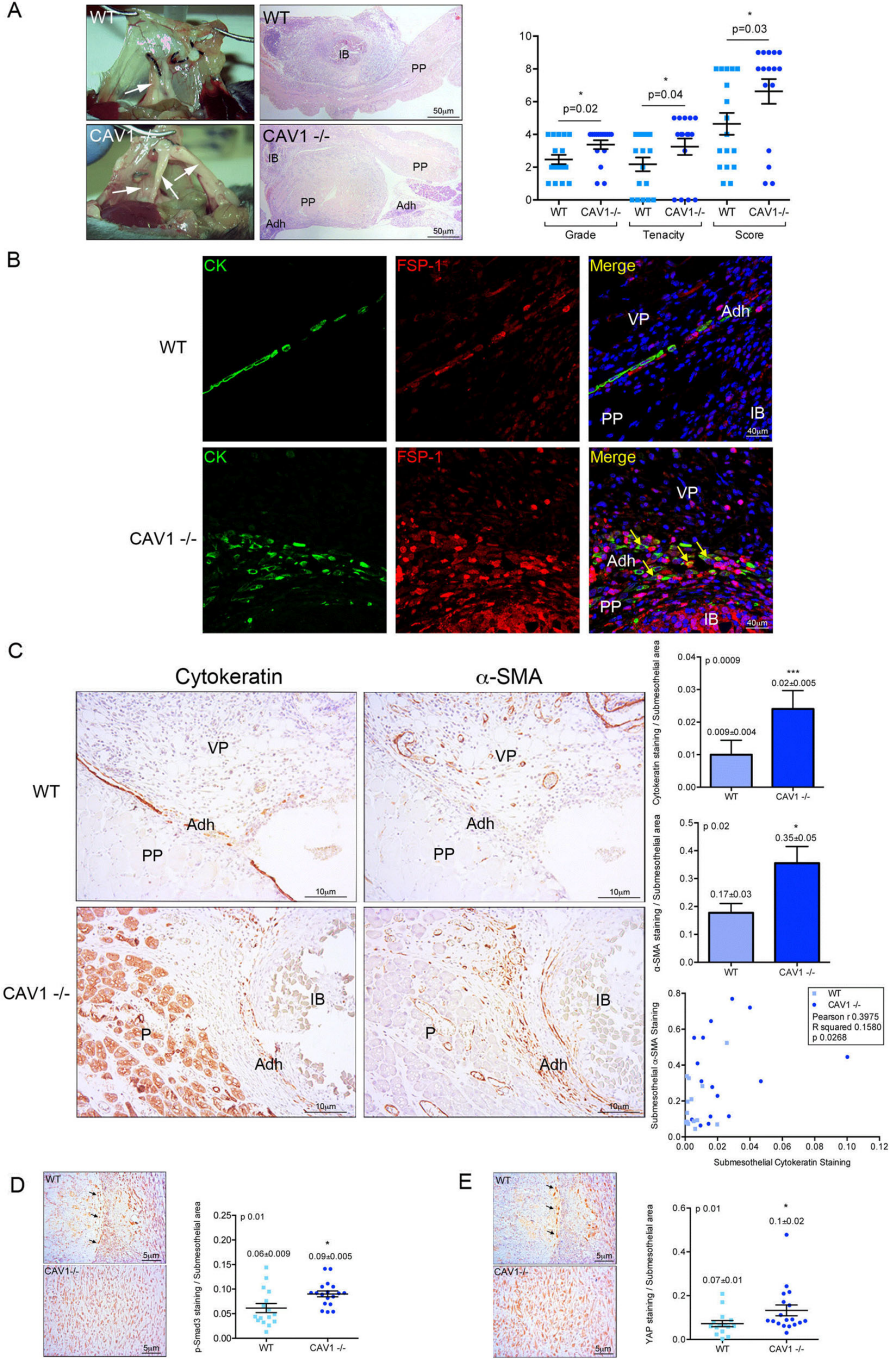
Exacerbated MMT and adhesion formation in a CAV1^−/−^ mouse model. **a** Upper left picture shows three IBs in a WT mouse. Arrow points to one adhesion scored as 6 (grade 2; tenacity 4). Lower left picture shows three adhesions (arrows) in a CAV1^−/−^ mouse scored as 6 (left; grade 2; tenacity 4), 7 (middle; grade 3; tenacity 4) and 9 (right; grade 4; tenacity 5), respectively. Upper right Haematoxylin&Eosin (H&E) image shows an IB without adhesion formation in a WT mouse. Lower right H&E image shows a parietal IB adhered to pancreas in a CAV1^−/−^ mouse. Quantification of adhesions shows significant differences between WT and CAV1^−/−^ mice. **b** Parietal tissues adjacent to IB areas were analyzed by double immunofluorescence for pan-cytokeratin (green) and FSP-1 (red). A representative picture of a WT mouse shows a mainly preserved MC monolayer in an IB adjacent area. Below, a representative image of a CAV1^−/−^ mouse shows double pan-cytokeratin/FSP-1 positive fibroblasts accumulated in the submesothelial zone of an adhesion. Nuclear 4′,6-diamidino-2-fenilindol (DAPI) is represented in blue. **c** Serial sections corresponding to IB areas were analyzed by immunohistochemistry for pan-cytokeratin and α-SMA. Representative pictures of a WT mouse show a preserved MC monolayer (pan-cytokeratin positive; α-SMA negative) in the adhesion zone. Serial tissue sections of a CAV1^−/−^ mouse show a severe adhesion area formed between parietal peritoneum and pancreatic tissue. Double pan-cytokeratin/α-SMA positive MCs are located into the submesothelial zone. Right graphics show submesothelial pan-cytokeratin and α-SMA staining quantifications. Submesothelial staining for pan-cytokeratin and α-SMA were significantly increased in CAV1^−/−^ compared to WT mice. Lower graphic shows a significant correlation between submesothelial pan-cytokeratin and α-SMA stains. **d** Peritoneal tissues were analyzed for phospho-Smad3 immunohistochemical staining, showing a significant increase of submesothelial positive areas in CAV1^−/−^ as compared to WT mice. **e** Similarly, YAP staining was significantly increased in submesothelial zones of CAV1^−/−^ as compared to WT mice. Graphic bars represent the mean ± SEM. Arrows point to the adhesion zone. IB ischemic button, PP parietal peritoneum, Adh adhesion, VP visceral peritoneum, P pancreas.

To study the implication of CAV1 in a mechanically induced peritoneal pathology, we induced the formation of PAs based on peritoneum ligation, taking advantage of an IB-based mouse model previously described^26^. Peritoneum ligation induced a rapid development of PAs, whose severity in terms of grade and tenacity was significantly increased in CAV1^−/−^ as compared to WT mice (Fig. 7a). Accordingly, immunofluorescence images depict pan-cytokeratin staining confined to a mainly preserved MC monolayer in an IB adjacent area of a WT mouse, while an increased submesothelial co-staining of both pan-cytokeratin and FSP-1 (a fibroblast marker) was found in adhesion zones of CAV1^−/−^ mice (Fig. 7b). Moreover, MMT was monitored by immunohistochemical analysis of IB serial sections for pan-cytokeratin and α-SMA (a marker of myofibroblast conversion). Peritoneal tissues from CAV1^−/−^ mice showed a significant increase in double-positive submesothelial areas compared to WT mice (Fig. 7c), as well as increase in phospho-SMAD3 and YAP staining (Fig. 7d, e). These results support a role of CAV1 as a key regulator of mechanically induced MMT and fibrosis induction during PA formation.

## Discussion

Due to its anatomical conformation and localization, the peritoneum is continuously exposed to biomechanical forces, such as those caused by diaphragm respiratory movements, physiological abdominal pressure or peristaltic movements. Abnormal mechanical cues may be originated by medical activities such as PD practice, due to increased abdominal pressure exerted by the large volumes (~2 L) of dialysis fluid infused into the peritoneal cavity of patients^49^. Interestingly, previous reports linked volume stress due to PD treatment to the production of endothelin-1, a vasoconstrictor peptide and transcriptional target of TGF-β1^50,51^. Other mechanical perturbations may arise from the trauma of the peritoneal membrane after abdominal laparotomies^26^. More recently, the accumulation of large volumes of ascitic fluid in patients with peritoneal carcinomatosis was linked to alterations of mechanical properties in the peritoneum^52^. Thus, although the hypothesis of a causal link between exposure to abnormal biomechanical forces and deveolpment of peritoneal fibrosis is coherent with reported indirect evidence, it has been scarcely explored experimentally so far.

Here, we demonstrate that exposure of peritoneal MCs to cyclic mechanical stretch is sufficient per se to induce a robust MMT phenotype. RNAseq and quantitative proteomics reveal mechanically induced activation of MMT, resembling previously described mesenchymal gene expression signatures in MCs associated with PD, peritoneal metastasis and experimental stimulation with TGF-β1 of primary MCs^27,39,46,53^. Accordingly, increasing evidence from mouse models and patient biopsies points to MMT as a major component of the development of post-surgical adhesions^4,26^. Mechanical stretching induces EMT in renal tubular epithelial cells, in lung epithelium and in keratinocytes^21–23^. However, mechanistic understanding at a molecular level of these relationships was limited and warranted the use of unbiased high-throughput techniques. To our knowledge, this is the first extensive characterization of MMT induction upon exposure to mechanical stretch.

Transcriptomic analysis revealed the upregulation of a TGF-β1 signaling signature in MCs exposed to mechanical stretch. When analyzing the functional contribution of TGF-β signaling, we found that MMT-compatible morphological changes and MMT-related genes including SNAI1, MMP2 and TGF-β1, were TGF-β signaling dependent. In fact, blocking TGF-β signaling was an efficient means to intervene MMT in a mouse model of PAs^26^. Importantly, the role of TGF-β1 signaling in mechanically induced EMT is likely cell type-specific, since it has been reported irrelevant in the progression of mechanically induced EMT in alveolar epithelial cells^21^.

YAP nuclear translocation depends on multiple factors, including extracellular factors, cell context parameters (i.e. local confluence) and substrate stiffness^13^. In our studies, exposure to mechanical stretch led to nuclear translocation of YAP and the modulation of the expression of both Hippo-YAP/TAZ network nodes and YAP transcriptional targets. In our in vitro experimental system, YAP was partially expressed in the nucleus in static conditions. This is in accordance with the basal nuclear staining observed in the mesothelial monolayer of healthy peritoneal human biopsies. It is conceivable that physiological cues, including mechanical stimuli such as physiological peristalsis, underpin this moderate basal activation of YAP. YAP/TAZ activation has already demonstrated to be causative to organ fibrosis in various organs, including kidney and lung and a cross talk between YAP/TAZ and TGF-β1 pathways has been described^11,54–58^. However, no reports to date have demonstrated a role of YAP/TAZ in the genesis of peritoneum fibrosis.

**Fig. 8:**
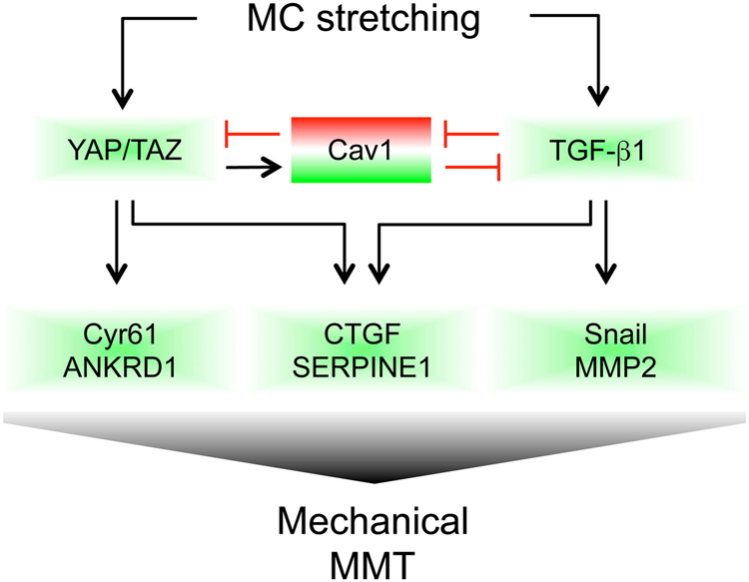
Interplay between TGF-β1 autocrine production and YAP/CAV1 activities mediates MMT induction and fibrosis upon exposure to cyclic mechanical stretch. Exposure to mechanical stretch promotes the activation of signaling pathways leading to increased TGF**-**β1 expression and induction of a MMT program. YAP/TAZ cooperates with TGF**-**β1 signaling in promoting the expression of genes involved in MMT induction such as CTGF, SERPINE1, ANKRD1, CYR61, SNAI1. YAP/TAZ supports also the expression of CAV1, which limits excessive MMT induction. Prolonged exposure to TGF**-**β1 downregulates CAV1 expression. In CAV1-silenced cells, TGF**-**β1 signaling is hyperactivated and YAP/TAZ specific genes are induced.

Our results also demonstrate that YAP/TAZ and TGF-β1 cooperate during mechanically induced MMT to regulate the expression of different subsets of genes. Expression of SERPINE1 and CTGF required both TGF-β1 and biomechanical pathways, whereas other genes, such as CYR61 and ANKRD1 seem to be mainly dependent on YAP/TAZ activity. Zanconato et al. demonstrated in static conditions the relevance of the association of YAP/TAZ/TEAD with AP-1 (whose activation may depend, at least in part, on TGF-β1 signaling) in driving cell growth^59^. According to these data, SMAD3 (a main TGF-β1 effector) has been demonstrated to interact with YAP, enhancing further trans-activation of the CTGF promoter^46^. Importantly, nuclear translocation of YAP by mechanical stimulation was largely refractary to the efficient blockade of TGF-β1 signaling, clearly demonstrating that TGF-β1 signaling does not directly impact mechanosensing upstream the YAP/TAZ system, but feeds onto the control of the transcriptional output of YAP/TAZ. Overall, this experimental evidence led us to elaborate a picture where: (i) Mechanical cues are transduced into mechanotransduction pathways, including YAP/TAZ pathway; (ii) Mechanical pathways lead to enhanced TGF-β expression/activity; and (iii) biomechanical and biochemical pathways cooperate during MMT induction (Fig. 8).

Interestingly, YAP was found to promote CAV1 expression during mechanical stretch. Our data linking CAV1 expression to YAP/TAZ activation are in agreement with a recent study demonstrating a direct binding of TEAD1 to CAV1 promoter^5^. We have previously demonstrated that CAV1 plays a role in maintaining the peritoneal homeostasis and its deficiency promotes MMT and fibrosis of the peritoneum during PD^12^. Herein, we found that CAV1-silenced MCs exposed to mechanical stretch show an exarcerbated dysregulation of MMT-related genes, a condition mainly characterized by a hyperactivation of TGF-β1 signaling. However, CAV1-dependent, TGF-β1 independent pathways promoting fibrosis cannot be excluded. The role of CAV1 in EMT appears to be cell/tissue specific, or stage/specific in tumours, since in other experimental systems CAV1 may in fact promote cellular EMT and invasion^8,60^. The synergism between CAV1 silencing and mechanical regulation of MMT-related genes, such as SNAI1 and CTGF, could be dependent on the activation of TGF-β1 pathway, since MMT features were strongly reduced upon exposure to a specific anti-TGF-β1 blocking monoclonal antibody. Mechanistically, our data showing a transcriptional regulation of TGF-β1 and TGFBRI expression by CAV1 deficiency may integrate previous reports dealing on the regulation of TGF-β1 signaling by CAV1-mediated endocytosis of TGFBRI^61,62^. Our experimental evidence suggests that CAV1 expression limits activation of profibrotic YAP target genes during MMT. CAV1/YAP interplay seems cell specific since evidence in other experimental systems clearly demonstrate that CAV1 positively regulates YAP^6^. It is unclear how CAV1 limits YAP activity in our experimental conditions. A role is played by the high levels of TGF-β1 production by MCs and by their high sensitivity to TGF-β. Interestingly, we found that TGFBRI expression is increased in CAV1-silenced and in CAV1^−/−^ MCs. In these conditions, enhanced TGF-β1 signalling may cooperate with YAP/TAZ in the transcriptional regulation of specific targets. Moreover, the alteration of cell junctions during MMT induced in the absence of CAV1 may play a role in favouring YAP nuclear translocation and activity, similarly to what was reported for other EMT experimental systems^58^.Overall, these data support the hypothesis of a negative feedback exerted by CAV1 in the process of mechanical MMT. In this condition, mechanical stretch-induced YAP activity promotes MMT-related changes, as well as CAV1 expression, which in turn exert a protective role over excessive TGF-β1 signaling. Interestingly, a prolonged activation of the TGF-β1 signaling may induce CAV1 downregulation, leading to an imbalance of this feedback regulation and promoting peritoneal fibrosis (Fig. 8).

Intra-abdominal adhesions are fibrous bands that tether organs to one another or to the parietal peritoneal wall, leading to a significant cause of post-surgical morbidity and posing a major public health challenge^63^. Their primary sequelae include bowel obstruction, female infertility, ectopic gestation, chronic abdominal and pelvic pain, poor quality of life, and death^64,65^. It is estimated that ~93% of patients undergoing abdominal surgery develop adhesions and ~20% require re-hospitalization. Our results confirm a role of MMT in PA formation as previously suggested^4,26^. MMT induction is particularly evident in the peritoneum of CAV1^−/−^ mice, in which transdifferentiated MCs appear as a major population invading the submesothelial stroma. In light of the data obtained from in vitro experiments, these results support a role of mechanical forces as an important driver of PA formation. Therefore, it is tempting to speculate that whereas biomechanical signals may be rather secondary in MMT during chronic peritoneal injuries (e.g. PD treatment), these stimuli could play a principal role in MMT during acute peritoneal damage (e.g. that occurring during abdominal surgery-related trauma). Nevertheless, the use of an IB mouse model to generate PAs limits the possibility of establishing a hierarchy of the different pathogenic forces implicated; besides the mechanical tension induced by IB ligation, hypoxia and coagulation may also play a role in the formation of the fibrotic response^4^.

In conclusion, our data reveal that exposure of the peritoneum to mechanical forces triggers a robust MMT response, in which the induction of biomechanical pathways cooperates with pathways induced by biochemical extracellular mediators, such as TGF-β1. Moreover, we elucidated complex peritoneal homeostatic responses elicited by the involvement of both CAV1 and YAP/TAZ pathway. Interfering MMT induced by mechanical injury may impact on the design of novel therapeutic opportunities to effectively prevent this pathological condition.

## Supplementary information

Supplementary figure and table legends

Supplementary figure 1

Supplementary figure 2

Supplementary figure 3

Supplementary figure 4

Supplementary figure 5

Supplementary figure 6

Supplementary figure 7

Supplementary table 1.

Supplementary table 2

Supplementary table 3

Supplementary table 4
