# Supplementary material for: Caveolin1 and YAP drive mechanically induced mesothelial to mesenchymal transition and fibrosis
Source: Cell Death Dis. 2020 Aug 3;11(8):647. doi: 10.1038/s41419-020-02822-1 (PMC7435273; doi:10.1038/s41419-020-02822-1)
Supplement: Supplementary file 1 — Supplementary figure and table legends [file 41419_2020_2822_MOESM1_ESM.docx]

**Supplementary Figures**

**Suppl. Fig. 1** HPMC purity was determinated by FACS analysis of podoplanin expression as standard mesothelial marker, and by ruling out any contamination with macrophages (CD45 positive cells).

**Suppl. Fig. 2** Gene Set Enrichment Analysis of HPMCs in stretching and static conditions. GSEA was applied to analyze the comparative transcriptomic profile of human peritoneal mesothelial cells in stretching and static conditions. **A.** GSEA plots for six selected gene sets, included in MSigDB databases "Oncogenic Signatures", "Chemical and Genetic Perturbations", "Reactome", and "Cellular Component Gene Ontology", annotated with normalized enrichment score (NES) and false discovery rate (FDR). Black vertical lines indicate the position of genes from each gene set along a list of 14374 genes ranked by logFC, represented by the red to blue gradient. Green curves represent the distribution of gene set genes along the ranked list. Positive or negative NES values indicate a relative accumulation of genes with positive or negative logFC, respectively, in stretching conditions relative to static. FDR values lower than 0.25 are taken as significant, in the context of GSEA. **B**. GSEA analysis for IPA derived gene sets. The left side of the panel represents genes with Cav1-dependent transcription or expression, derived from the literature, according to IPA knowledgebase. Positively and negatively regulated genes, represented with orange and blue symbols, respectively, were used to define two gene sets: CAV1_POS_REG and CAV1_NEG_REG. GSEA was then used to determine whether any of the two gene sets was enriched among genes up-regulated or down-regulated upon stretching. GSEA results, presented in the middle and right sections of the panel, indicated that Cav1 negatively regulated genes are enriched among the genes that are up-regulated upon stretching and that enrichment for CAV1_NEG_REG is significant (NES = 1.59; FDR = 0.028). SNAI1, CSF1R and CCND1 are the genes that mostly contribute to the observed enrichment value (they are the leading-edge subset of genes, as defined for GSEA), and their relative expression values are presented in the heatmap below. At the same time, Cav1 positively regulated genes (the CAV1_POS_REG gene set) are significantly enriched among the genes that are down-regulated upon stretching (NES = -1.25; FDR = 0.15). The leading-edge subset of genes is composed of NFATC1, CD14, NR3C2, FOS and EGR1 and their relative expression values are presented in the heatmap below. **C.** Graphical summary of results. The barplot represents FDR values for the eight gene sets referred in panels A and B. The dashed lines, representing FDR values of 0.25 (blue) and 0.05 (red), indicate the significance threshold used by default in the context of GSEA, and a more strict significance threshold, respectively. Positive and negative NES values are represented by colour ranging from green to brown.

**Suppl. Fig. 3** IPA network representing interactions between genes shown in **Fig. 2B** and with YAP, TAZ, CAV1, TGF-β1 and EMT function. Continuous lines represent direct relationships. Dotted lines represent indirect interactions. Significantly upregulated genes are in green. Significantly downregulated genes are represented in red.

**Suppl. Fig. 4** IPA Network representing interactions between genes shown in **Fig. 6A** and with Cav1, TGF-β1 and EMT function. Continuous lines represent direct relationships. Dotted lines represent indirect interactions. Significantly upregulated genes are in green. Significantly downregulated genes are represented in red.

**Suppl. Fig. 5** qRT-PCR using two different siRNA sequences specific for CAV1. In both cases, CAV1 silencing in stretching conditions (as in **Fig. 6B**) further promotes induction of mesenchymal markers compatible with a bona fide MMT. L34 mRNA levels were used for normalization. Bars represent the mean ±SEM of duplicate determinations in five to six independent experiments.

**Suppl. Fig 6.** Additional statistical comparisons from **Fig. 5A.**

**Suppl. Fig 7.** Additional statistical comparisons from **Fig. 6B.**

**Supplementary Tables**

**Suppl. Table 1.** Primer pairs for qRT-PCR used in this study.

**Suppl. Table 2.** Post-surgical adhesion mouse scoring scheme. Grade is the extent of the IB covered by the adhesion, represented as a percentage.

**Suppl. Table 3.** A list of mesothelium-specific markers was created on the basis of published transcriptomic studies. KRT7, calretinin, podoplanin, mesothelin and WT1 have been considered as specific mesothelial markers^48^. Ruiz Carpio et al identified profiles of gene expression of MCs during in vivo and ex vivo MMT^39^. Moreover, previous research demonstrates that MCs typically share common markers with endothelial cells, including VEGF, VEGF receptors (KDR) and co-receptors (neuropilin-1). Accordingly, VEGF/VEGFRs/co-receptors axis switched during MMT^47^. A number of independent analyses from other studies further confirm our data^51^.

**Suppl. Table 4.** List of Cav1 and TGF-β1 related genes from the experiment described in **Fig. 6**. Upregulated genes are in green; downregulated genes are in red. p values ≤ 0.05 are highlighted in blue.
