# Supplementary figures and images for: Caveolin1 and YAP drive mechanically induced mesothelial to mesenchymal transition and fibrosis
Source: Cell Death Dis. 2020 Aug 3;11(8):647. doi: 10.1038/s41419-020-02822-1 (PMC7435273; doi:10.1038/s41419-020-02822-1)

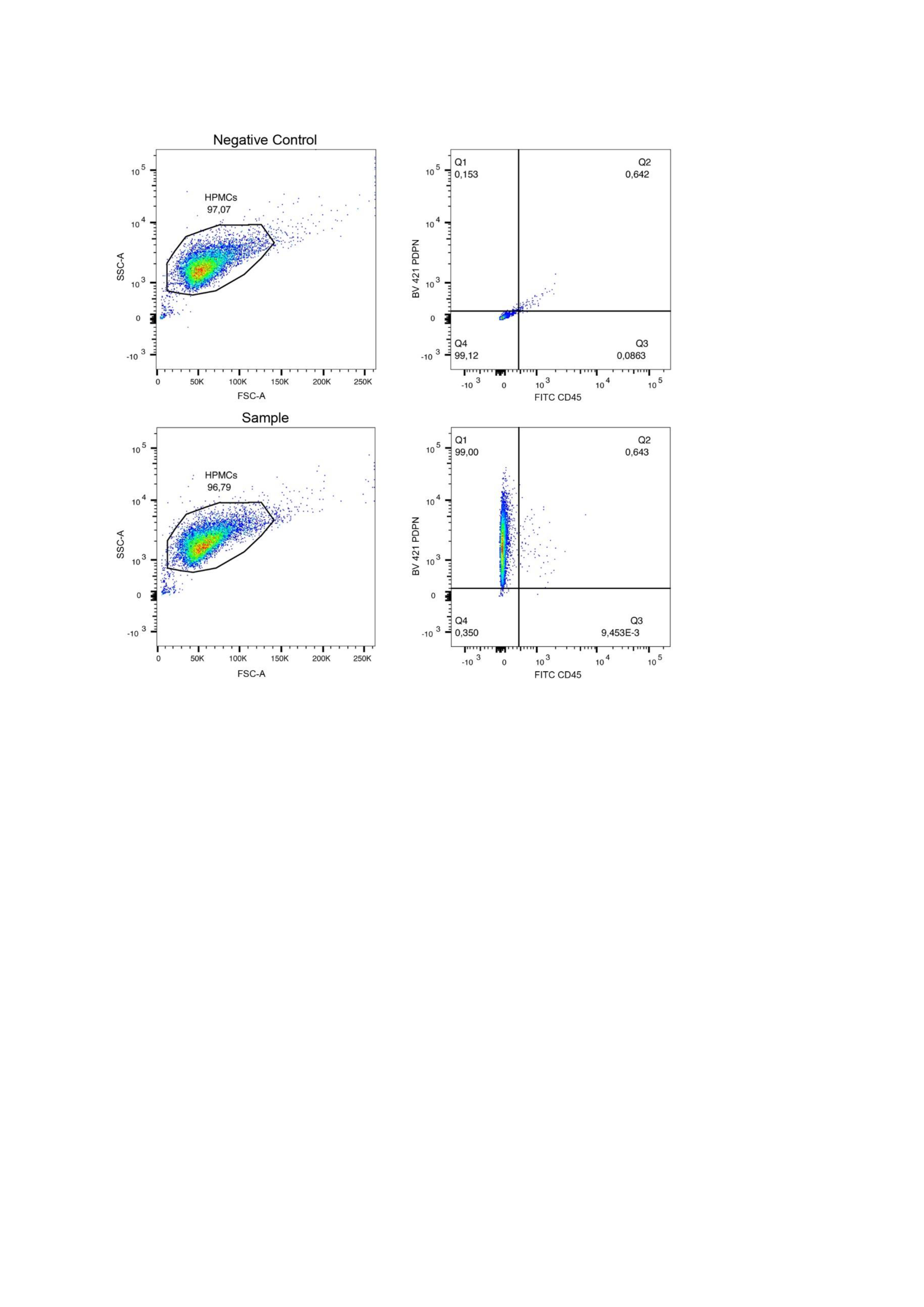

Supplement: Supplementary file 2 — Supplementary figure 1 [file 41419_2020_2822_MOESM2_ESM.tif]

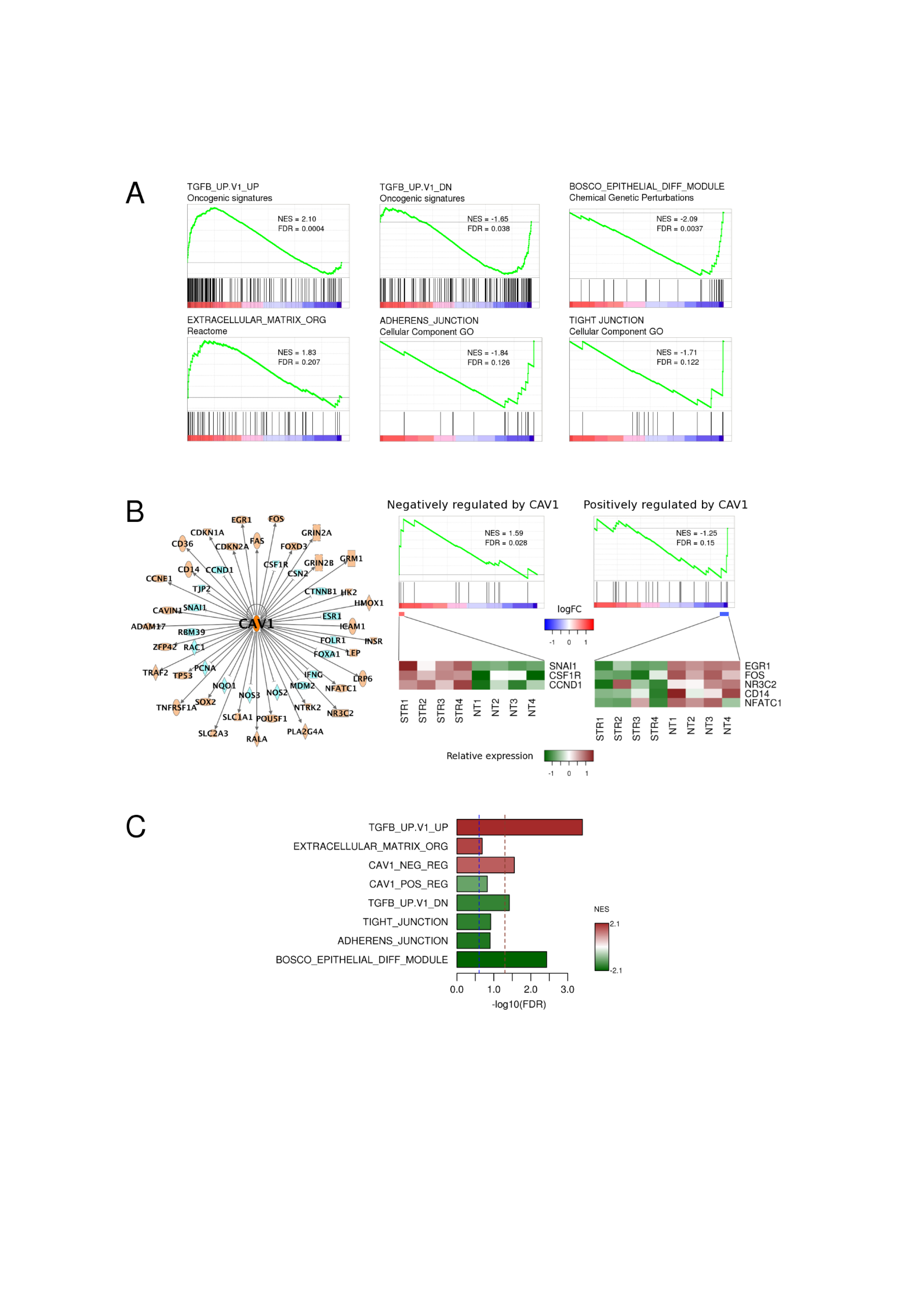

Supplement: Supplementary file 3 — Supplementary figure 2 [file 41419_2020_2822_MOESM3_ESM.tif]

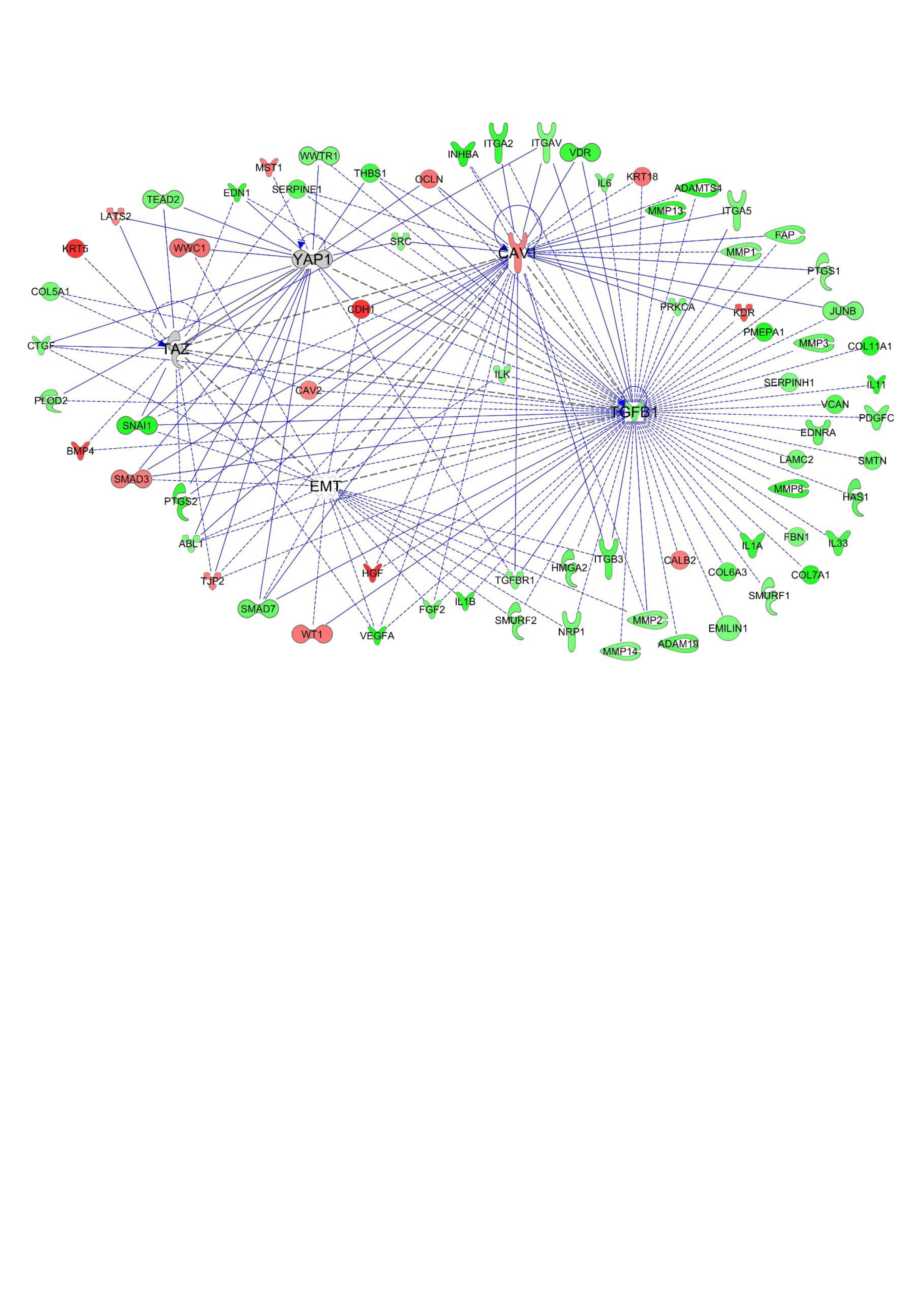

Supplement: Supplementary file 4 — Supplementary figure 3 [file 41419_2020_2822_MOESM4_ESM.tif]

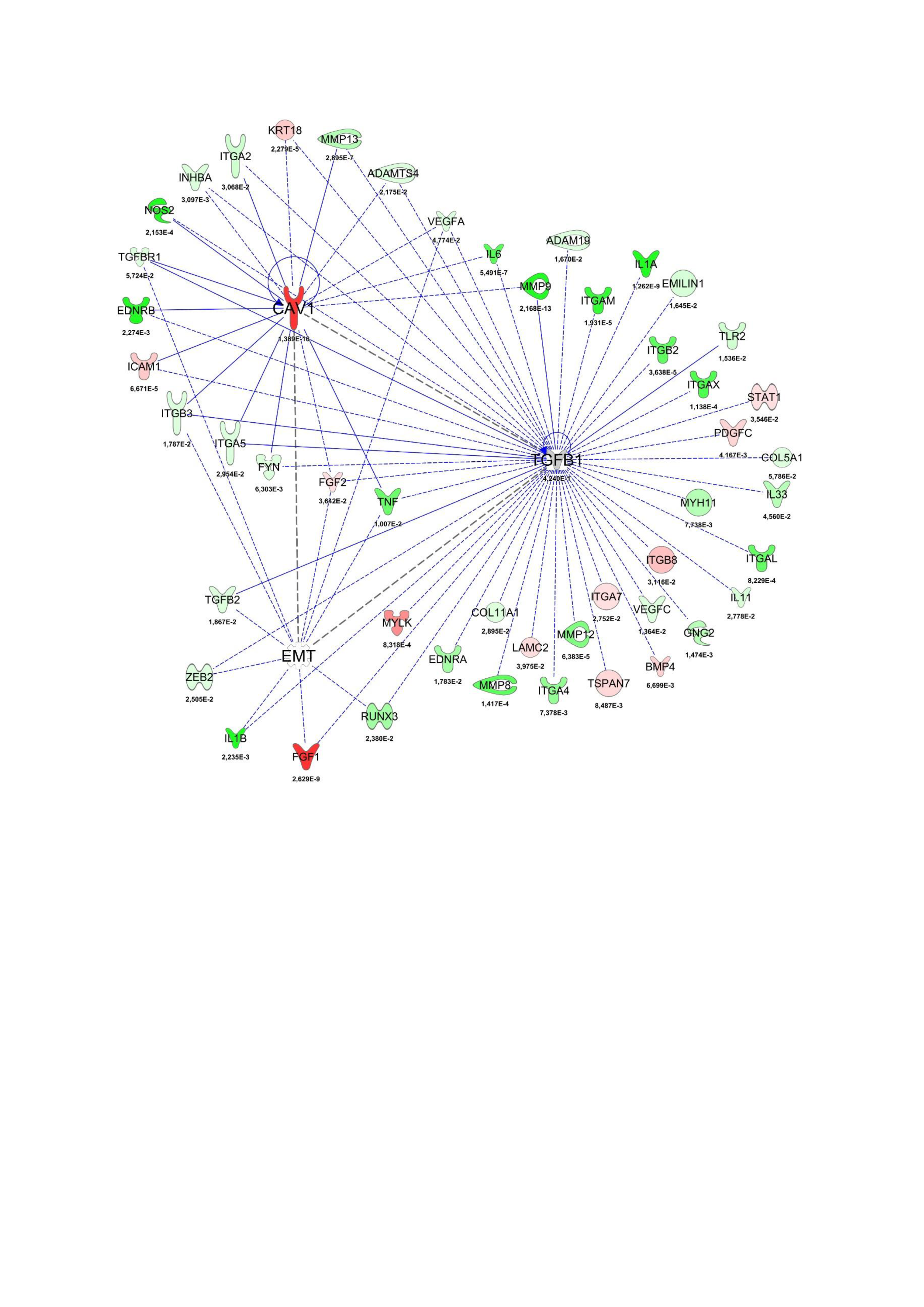

Supplement: Supplementary file 5 — Supplementary figure 4 [file 41419_2020_2822_MOESM5_ESM.tif]

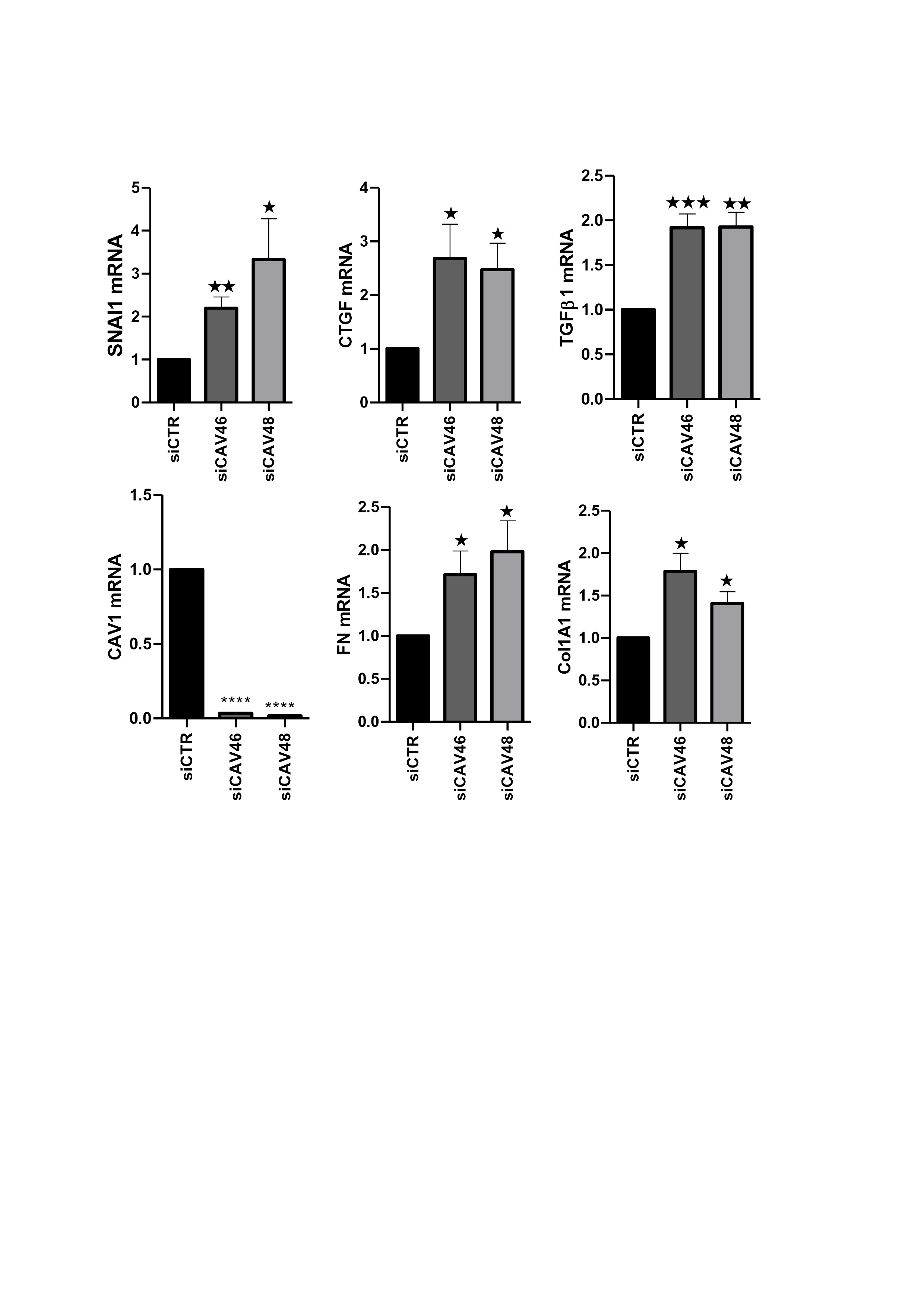

Supplement: Supplementary file 6 — Supplementary figure 5 [file 41419_2020_2822_MOESM6_ESM.tif]

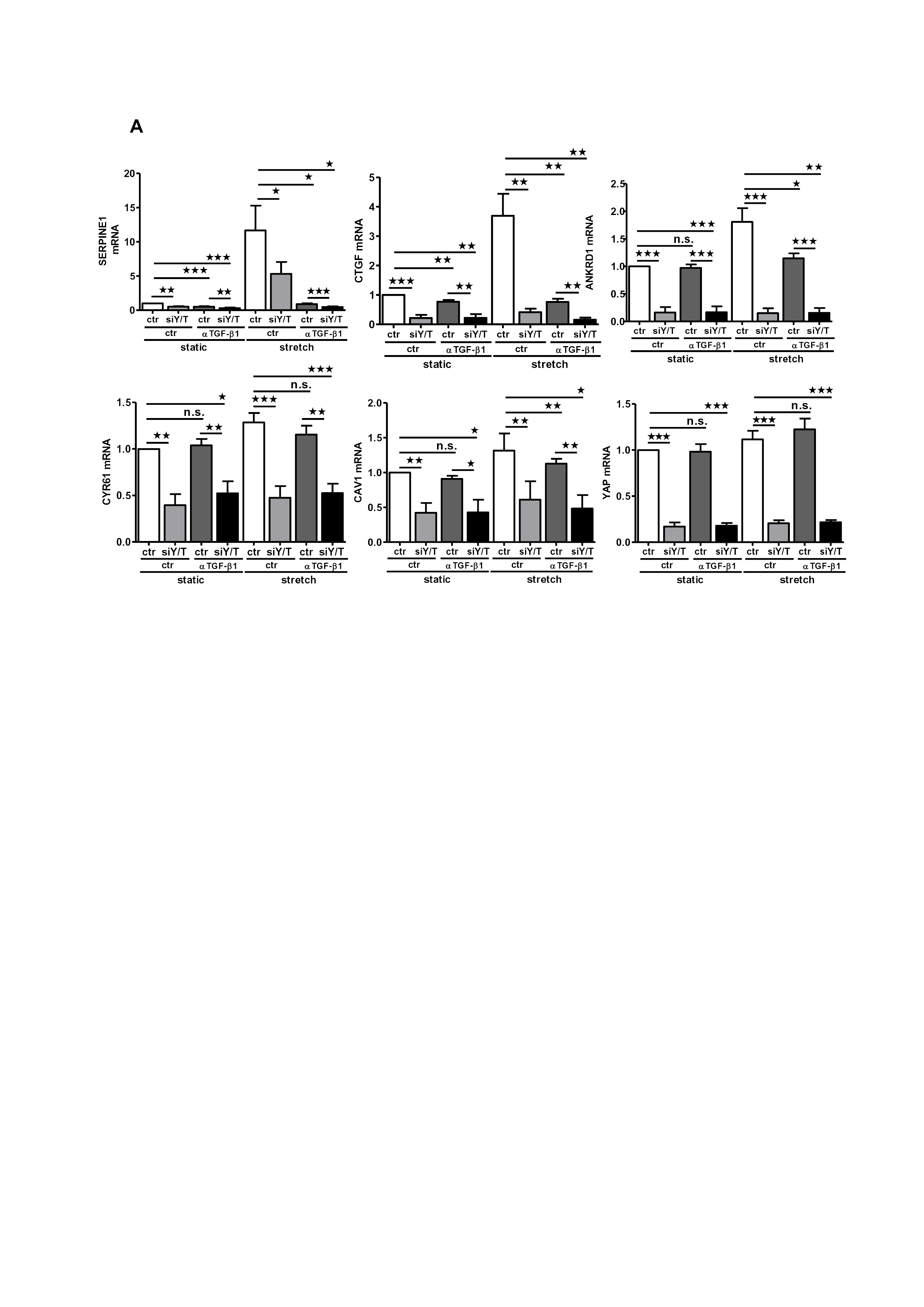

Supplement: Supplementary file 7 — Supplementary figure 6 [file 41419_2020_2822_MOESM7_ESM.tif]

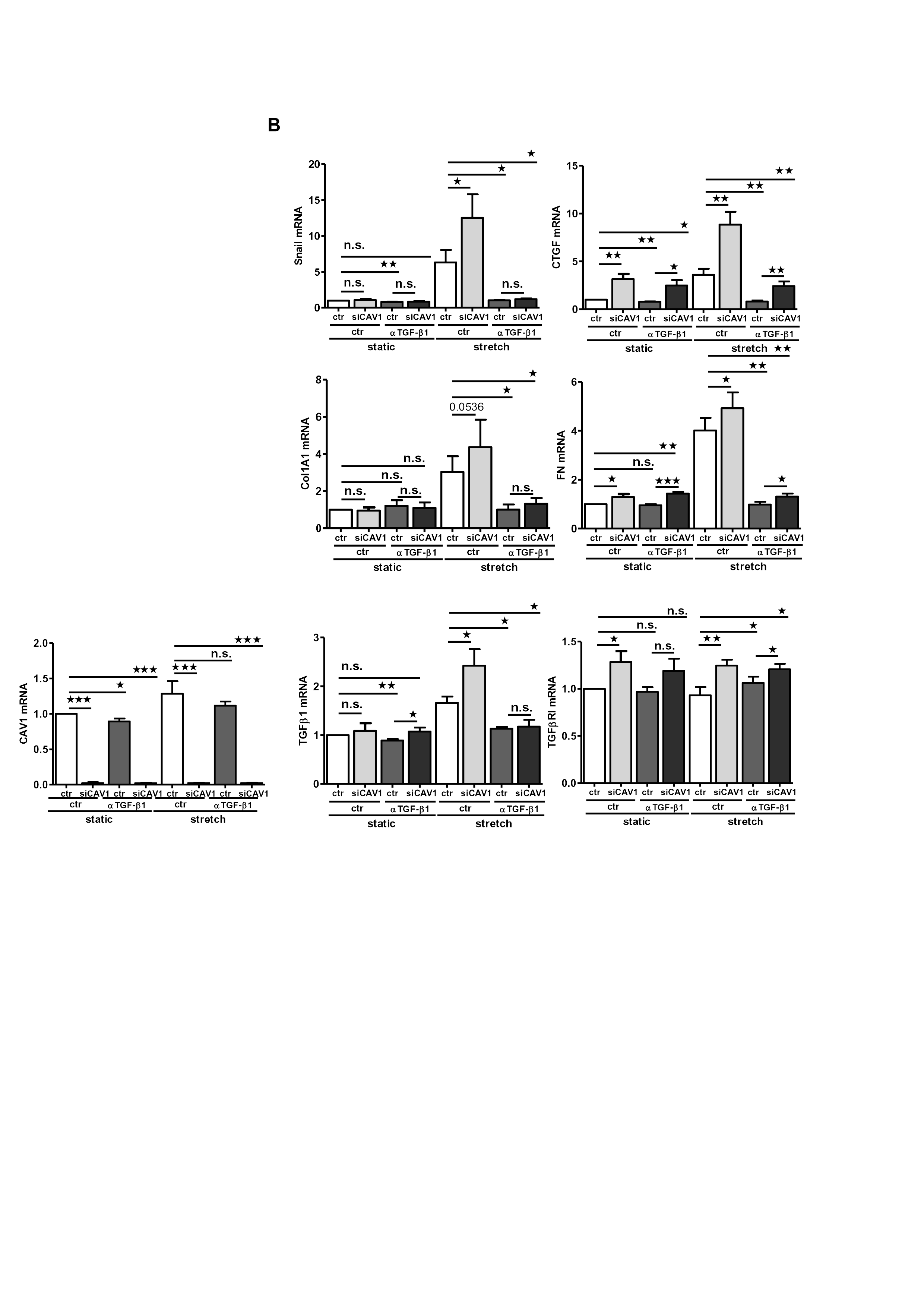

Supplement: Supplementary file 8 — Supplementary figure 7 [file 41419_2020_2822_MOESM8_ESM.tif]

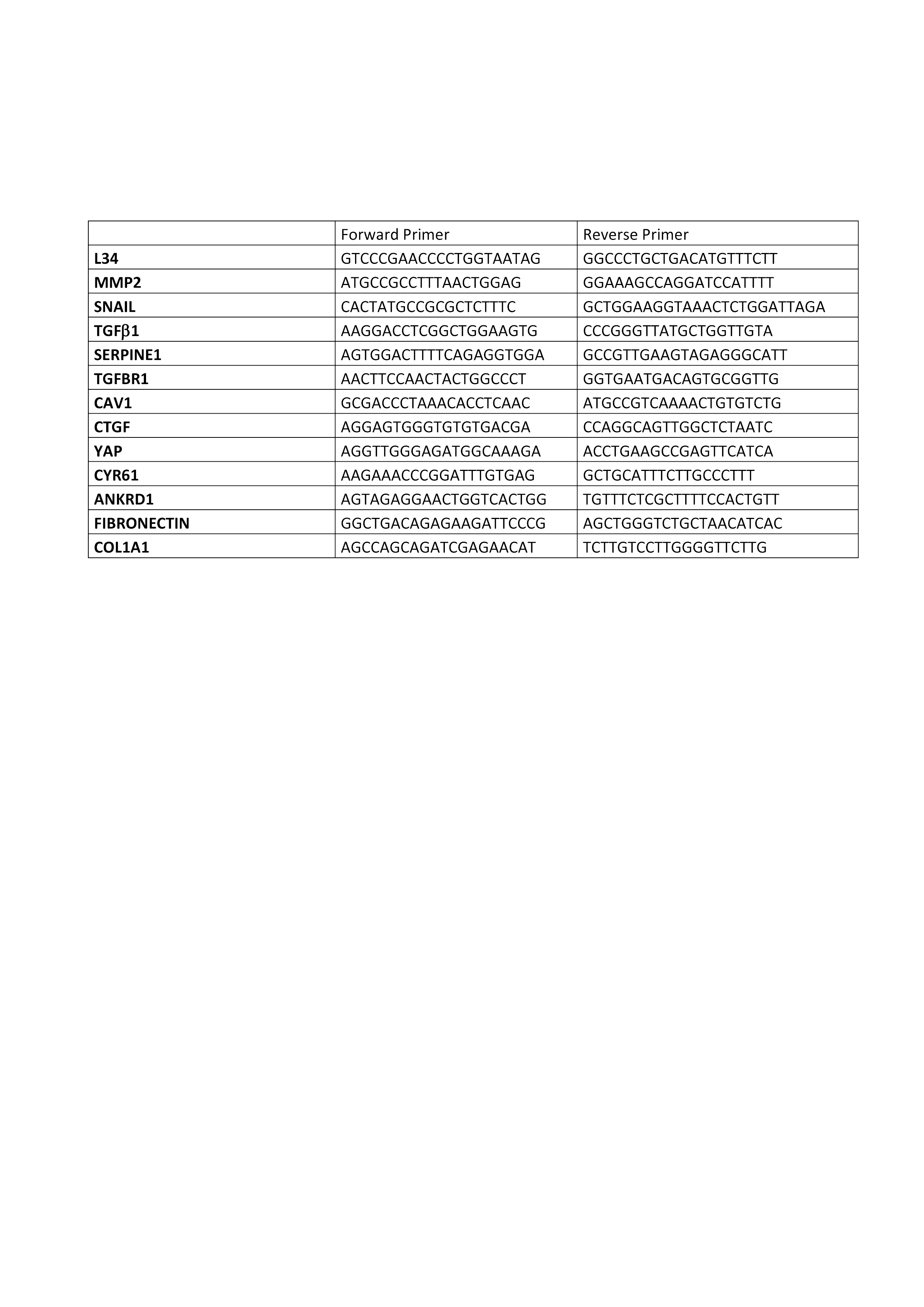

Supplement: Supplementary file 9 — Supplementary table 1. [file 41419_2020_2822_MOESM9_ESM.tif]

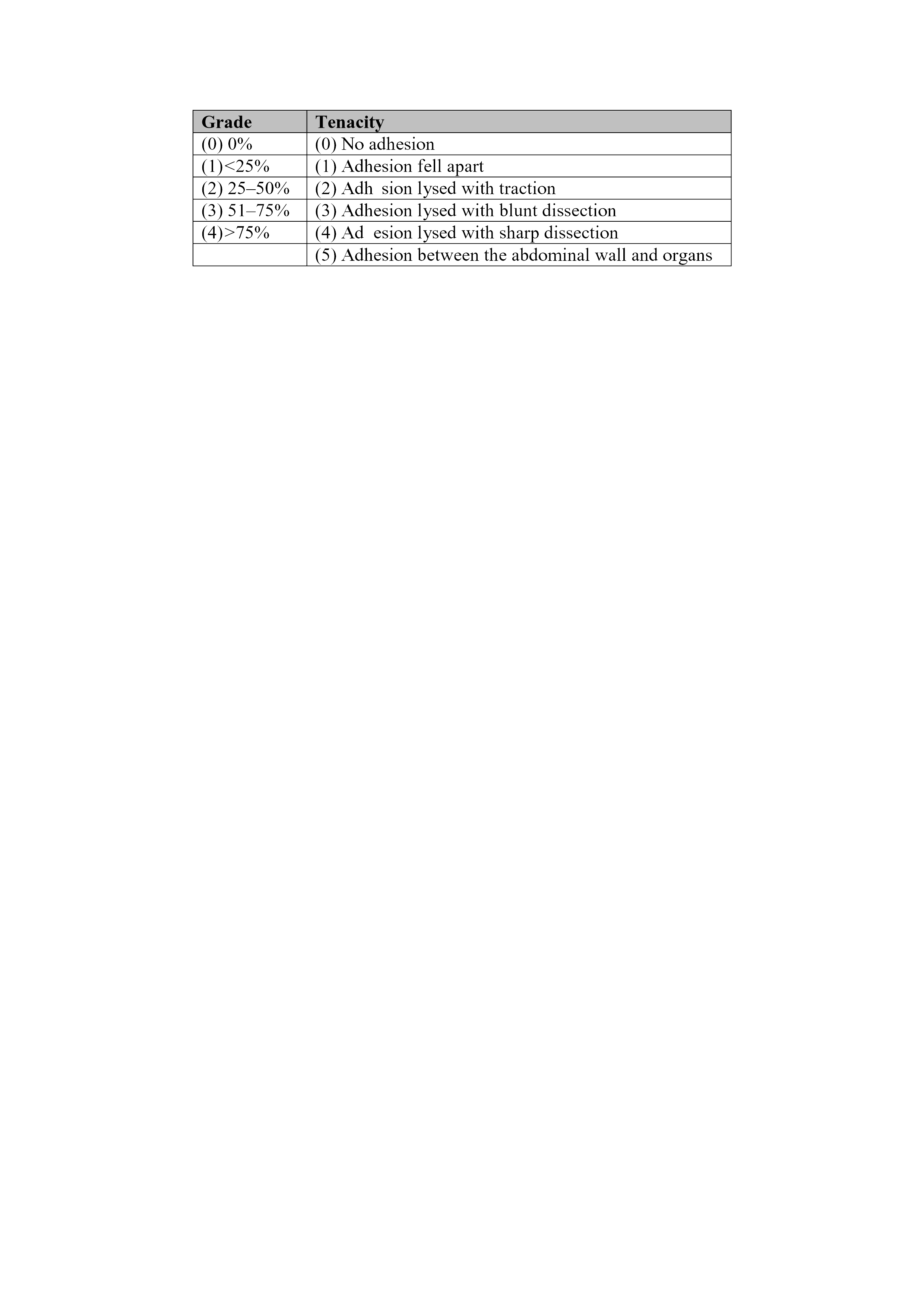

Supplement: Supplementary file 10 — Supplementary table 2 [file 41419_2020_2822_MOESM10_ESM.tif]

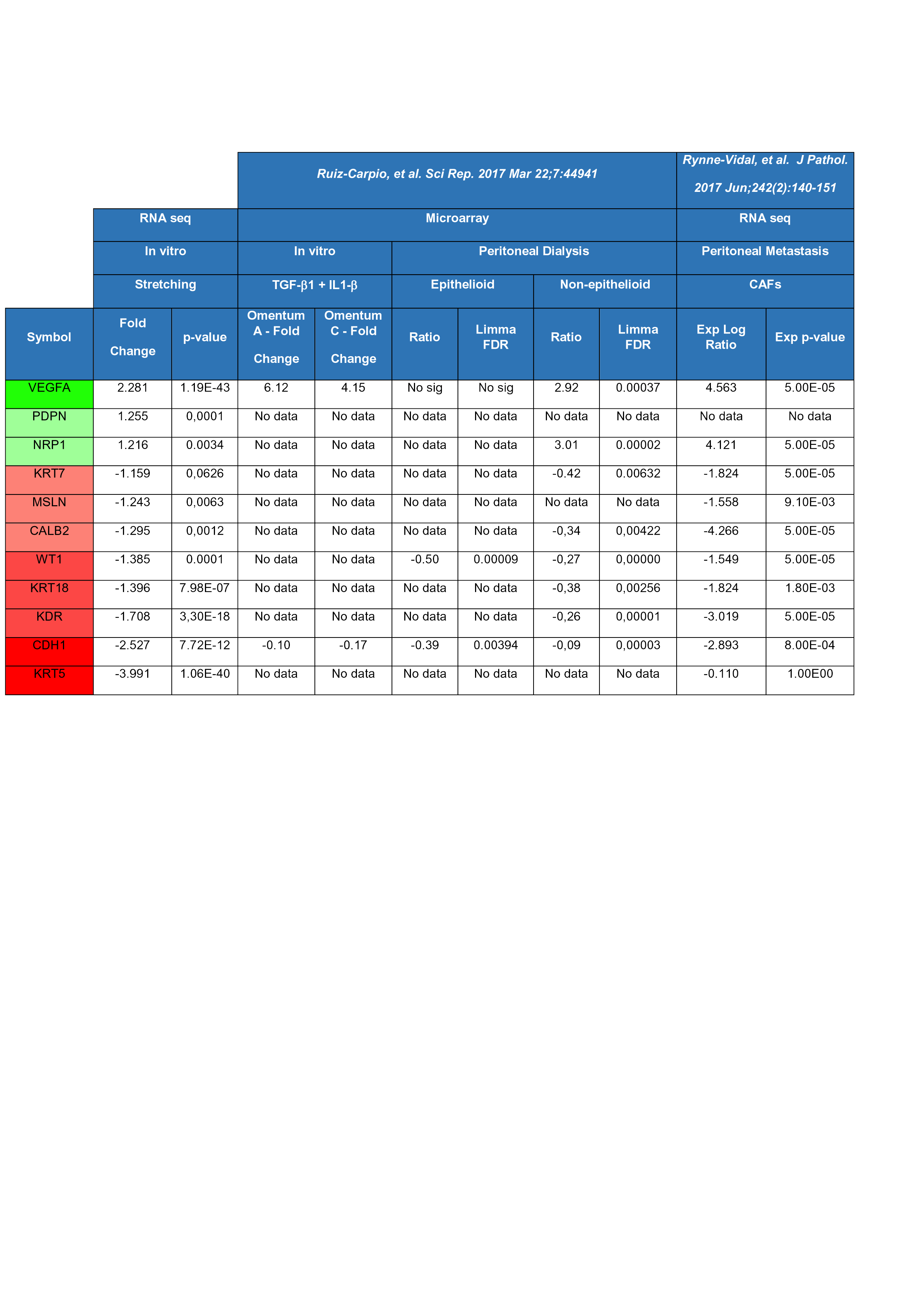

Supplement: Supplementary file 11 — Supplementary table 3 [file 41419_2020_2822_MOESM11_ESM.tif]

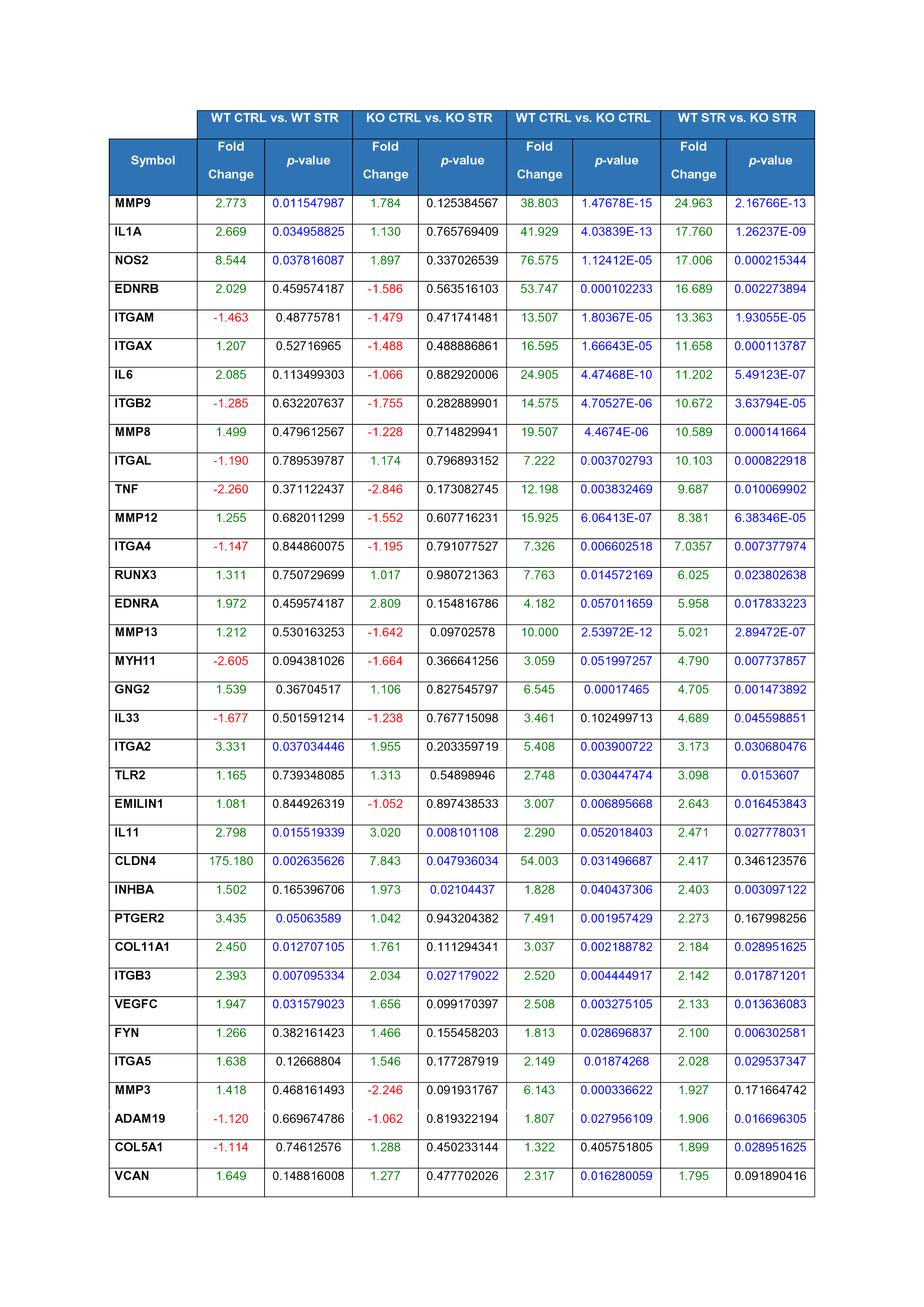

Supplement: Supplementary file 12 — Supplementary table 4 [file 41419_2020_2822_MOESM12_ESM.tif]
